# Functional Characterization and Mutagenesis Studies of a Microbial-like Diterpene Synthase from *Huperzia serrata*

**DOI:** 10.3390/molecules31081329

**Published:** 2026-04-17

**Authors:** Ting He, Yao Zhao, Xin Li, Bao Chen, Fangyan Chen, Baofu Xu

**Affiliations:** 1 School of Pharmaceutical Sciences, Shandong University of Traditional Chinese Medicine, Jinan 250355, China; 18291650451@163.com; 2 Shandong Laboratory of Yantai Drug Discovery, Bohai Rim Advanced Research Institute for Drug Discovery, Yantai 264117, China; shxyz4@nottingham.edu.cn (Y.Z.); xli@baridd.ac.cn (X.L.); bchen@baridd.ac.cn (B.C.); 3 Shanghai Institute of Materia Medica, Chinese Academy of Sciences, Shanghai 201203, China; 4 Nottingham Ningbo China Beacons of Excellence Research and Innovation Institute, University of Nottingham Ningbo, Ningbo 315100, China

**Keywords:** *H. serrata*, MTPSL, multifunctional, mutation, spatane, P450

## Abstract

Over the past decade, an increasing number of functional microbial-like terpene synthases (MTPSLs) have been reported in non-seed plants. However, whether the traditional Chinese medicinal plant *H. serrata* harbors such enzymes and their corresponding functions remains unexplored. In this study, we mined the transcriptome of *H. serrata* and identified a microbial-like terpene synthase, *Hs*MTPSL1, which produces multiple diterpene products. Following isolation and structural elucidation, seven distinct compounds were obtained, representing three skeletal types: spatane, prenylkelsoene-type, and biflorane. Among these, compound **7** is a novel biflorane diterpene. Structural analysis and subsequent mutagenesis revealed critical residues governing the formation of distinct skeletons, uncovering the multifunctional nature of this enzyme. Notably, the S224A mutation significantly enhanced the production of spatane diterpene compound **1** by 11.6-fold, demonstrating the potential for protein engineering to improve the yield of this bioactive marine-specific diterpene. Transcriptomic profiling revealed that *Hs*MTPSL1 is highly expressed in sporangia, and co-expression analysis with cytochrome P450s identified the CYP781 subfamily as candidates potentially involved in the downstream modification of these skeletons. Collectively, we report the first MTPSL from *H. serrata* and characterize it as a multifunctional diterpene synthase. Through structure-guided mutagenesis, we uncovered the molecular basis of its functional versatility, with the S224A mutation providing a powerful tool for enhancing the yields of all three diterpene skeletons, thereby laying a foundation for future protein engineering and synthetic biology applications.

## 1. Introduction

*Huperzia serrata*, a traditional Chinese medicinal plant belonging to the lycophyte family, has long been recognized for its rich repertoire of bioactive natural products. Among these, the alkaloid huperzine A has garnered significant attention as a potent acetylcholinesterase inhibitor for the treatment of Alzheimer’s disease [1,2]. In addition to alkaloids, *H. serrata* accumulates a diverse array of terpenoid constituents, including triterpenoids [3] and diterpenoids (3*β*-hydroxy-isopimaric acid, dehydroabietic aldehyde, and pisiferic acid, etc.) [4,5], many of which are thought to contribute to the plant’s pharmacological properties [5,6]. Despite the well-documented chemical diversity of terpenoids in this species, the enzymatic machinery responsible for their biosynthesis, particularly the terpene synthases that generate the complex hydrocarbon scaffolds, remains largely unexplored [7]. Uncovering these enzymes is essential for understanding the biosynthetic logic underlying terpenoid diversity in this medicinally important plant and for enabling future metabolic engineering efforts.

In non-seed plants, a phylogenetically distinct clade of terpene synthases known as microbial-like terpene synthases (MTPSLs) has emerged as a key source of biosynthetic novelty [8,9,10]. First characterized in the lycophyte *Selaginella moellendorffii*, MTPSLs are evolutionarily closer to bacterial terpene synthases than to canonical plant terpene synthases, and they have been shown to produce structurally diverse and sometimes unprecedented terpene scaffolds [11,12,13]. The presence of MTPSLs in early-diverging plant lineages suggests that these enzymes may represent an ancient reservoir of catalytic diversity, capable of generating complex terpene skeletons that are not commonly found in angiosperms. However, the functional repertoire of MTPSLs in *H. serrata* and their potential roles in the biosynthesis of its diverse terpenoid constituents have not been systematically investigated.

In this study, we report the discovery and functional characterization of *Hs*MTPSL1, an MTPSL from *H. serrata* identified through transcriptome mining. Heterologous expression revealed that *Hs*MTPSL1 is a multifunctional diterpene synthase capable of producing seven distinct diterpenes belonging to three different skeletal types: spatane, prenylkelsoene-type, and biflorane. Notably, the identification of spatane diterpenes from a plant source is unprecedented, challenging the long-held view of this scaffold as exclusive to marine organisms. Through structure-guided mutagenesis, we identified key residues governing the formation of each skeletal type, uncovering the molecular basis for the enzyme’s functional versatility. Phylogenetic analysis placed *Hs*MTPSL1 in a clade closer to bacterial terpene synthases than to higher plant TPSs, while sequence alignment revealed lineage-specific residues that fine-tune product partitioning among the three skeletal types. Furthermore, transcriptomic profiling showed that *Hs*MTPSL1 is highly expressed in sporangia and co-expressed with cytochrome P450s of the CYP781 family, implicating them in downstream oxidative modifications. Collectively, our findings establish *Hs*MTPSL1 as the first functionally characterized MTPSL from *H. serrata*, expand our understanding of terpenoid biosynthetic diversity in early-diverging plants, and provide a foundation for future protein engineering and natural product discovery.

## 2. Results

### 2.1. Functional Characterization of a Microbial-like Terpene Synthase from H. serrata

**Figure 1:**
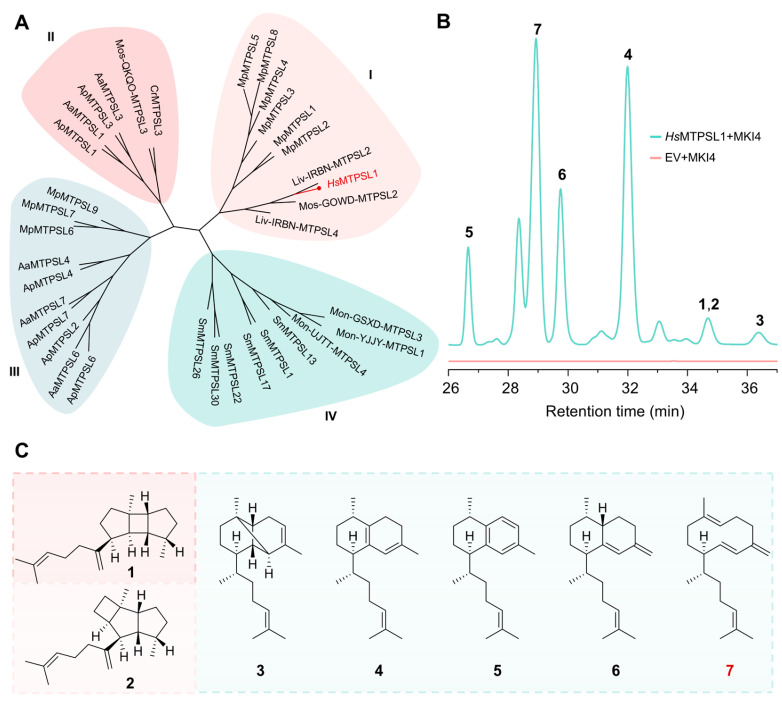
Functional characterization of *Hs*MTPSL1. (**A**) Phylogenetic analysis of *Hs*MTPSL1 with selected MTPSLs. The neighbor-joining tree was constructed based on amino acid sequences of MTPSLs from non-seed plants (Table S3) and *Hs*MTPSL1 (highlighted in red) clusters within Group I MTPSLs. (**B**) HPLC analysis of *Hs*MTPSL1 enzyme activity using the GGPP overproduction system CDF-MKI4. Cell extracts of *E. coli* expressing pET28a-*Hs*MTPSL1 together with pCDF-MKI4 were analyzed by reverse-phase HPLC with UV detection at 210 nm (blue trace). The empty vector control pET28a with pCDF-MKI4 (red trace) shows no corresponding peaks. Seven distinct diterpene products (**1**–**7**) were isolated, with retention times indicated. (**C**) Chemical structures of compounds **1**–**7**. Structures were determined by NMR and GC-MS. Compounds **1**–**6** are known diterpenes, while compound **7** is a new diterpene possessing a prenylgermacrane skeleton. Compound **1** is a spatane skeletal diterpene, compound **2** is a prenylkelsoene-type diterpene, and compounds **4**–**6** belong to the biflorane skeletal diterpenes.

In our ongoing search for novel microbial-like terpene synthases (MTPSLs) in non-seed plants [13], we screened the transcriptome of *H. serrata* [14] using the annotation “microbial-like terpenoid synthase”. A candidate gene, designated *Hs*MTPSL1, was identified via SwissProt annotation. Phylogenetic analysis with reported MTPSLs classified *Hs*MTPSL1 into group I, a clade previously associated with bacterial-type terpene cyclization functions [9] (Figure 1A). The codon-optimized gene was synthesized and expressed in *E. coli* using a pET28a vector, which was subsequently introduced into a GGPP overproducing chassis strain (CDF-MKI4 overexpressed) [15] for activity screening. Initial assays indicated low enzymatic activity, prompting us to enhance soluble expression by truncating the gene and fusing it with an acidic short peptide (CC-Di-A) [16]. HPLC analysis of the recombinant enzyme assay revealed that *Hs*MTPSL1 produces multiple diterpenoid metabolites. Following large-scale fermentation, separation, and purification, seven compounds were obtained, and their structures were elucidated by NMR spectroscopy (Figure 1B and Figures S1–S20). Among these, compounds **1**–**6** were identified as known diterpenes [15,17,18,19,20], whereas compound **7** was a novel diterpene featuring a prenylgermacrane skeleton [21] (Figure 1C and Table S1). Compounds **4**–**6** also belong to the biflorane skeletal type, and compound **2** was identified as a prenylkelsoene-type diterpene. Notably, compound **1** was a spatane diterpene.

The spatane diterpenoids are characterized by a unique *cis*,*anti*,*cis*-tricyclo[5.3.0.0]decane ring system, representing a highly strained bridged-ring architecture [22]. These compounds are primarily distributed in marine organisms, such as corals [21,23,24] and brown algae [25,26], and are recognized as typical marine natural products. Since the initial isolation of spatol from *Spatoglossum schmittii* in the 1980s [27], a series of spatane derivatives have been identified and characterized, many of which exhibit promising biological activities including cytotoxicity, anti-inflammatory, and antifouling properties [23,26,28,29]. Remarkably, compound **1** represents the first spatane diterpene identified from a plant source, and *Hs*MTPSL1 is the first terpene synthase reported in plants capable of generating this scaffold—notwithstanding its relatively low abundance among the *Hs*MTPSL1 products, accounting for only approximately 0.24% of the seven isolated compounds (Figure S21), with a yield of 0.19 mg/L (Figures S22 and S23 and Table S2). These results establish *Hs*MTPSL1 as a multifunctional diterpene synthase capable of generating a diverse product profile that includes the characteristic marine-associated spatane core.

**Figure 2:**
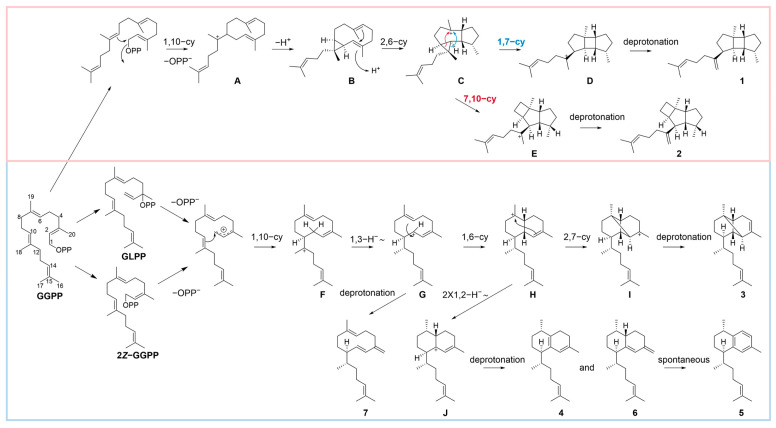
The presumed potential biosynthetic pathway. GGPP undergoes initial 1,10-cyclization to form intermediate **A**. Deprotonation and cyclopropane ring formation give neutral intermediate **B**, which, upon reprotonation at C-3, triggers a second 2,6-cyclization to generate cation **C**. From **C**, two alternative cyclopropane ring-opening routes lead to spatane skeletal compound **1** (via 1,7-cyclization to intermediate **D**) and prenylkelsoene-type compound **2** (via 7,10-cyclization to intermediate **E**). Alternatively, GGPP is first isomerized to GLPP or 2*Z*-GGPP, followed by 1,10-cyclization to produce intermediate **F** and a 1,3-hydride shift to produce intermediate **G**. Deprotonation of **G** yields monocyclic compound **7**; further 1,6-ring closure gives intermediate **H**. From **H**, 2,7-cyclization generates intermediate **I**, which upon deprotonation affords tricyclic compound **3**, whereas two successive 1,2-hydride shifts convert **H** to intermediate **J**, a precursor of bicyclic compounds **4** and **6**. Spontaneous oxidation of **4** and **6** may generate compound **5**.

Based on the seven products isolated from *Hs*MTPSL1, we proposed a biosynthetic pathway for these compounds. Drawing on the established mechanism of *Sx*SpS from *Streptomyces xinghaiensis* for the formation of tricyclic compounds **1** and **2** [17], we suggest that the biosynthesis of **1** and **2** proceeds via 1,10-cyclization to intermediate **A**, followed by deprotonation to form a cyclopropane ring, yielding the neutral intermediate **B**. Reprotonation at C-3 of **B** then leads to a second 2,6-cyclization, generating cation **C**. This cation can undergo two alternative cyclopropane ring-opening pathways, either via 1,7-cyclization to afford intermediate **D**, the precursor of **1**, or via 7,10-cyclization to give intermediate **E**, the direct precursor of **2**. Regarding the monocyclic compound **7**, bicyclic compounds **4**–**6**, and tricyclic compound **3**, their formation is proposed to be analogous to that catalyzed by *Pc*TS1 from *Paramuricea clavate* [15], as they all yield the same elisabethatriene (**6**) product. In this pathway, GGPP is first isomerized within the enzyme’s active site to geranyllinalyl diphosphate (GLPP) or 2*Z*-GGPP, introducing a 2*Z* double bond essential for subsequent cyclization. A 1,10-cyclization followed by a 1,3-hydride shift produces intermediate **G**, which can undergo deprotonation to give **7**. Intermediate **G** further undergoes a 1,6-ring closure to form **H**. From **H**, a 2,7-cyclization with deprotonation leads to **3**. Alternatively, **H** can undergo two successive 1,2-hydride shifts to yield intermediate **J**, a potential precursor of **4** and **6**. Compounds **4** and **6** may then undergo spontaneous oxidation to form compound **5** (Figure 2).

### 2.2. Docking and Mutational Analysis of HsMTPSL1

**Figure 3:**
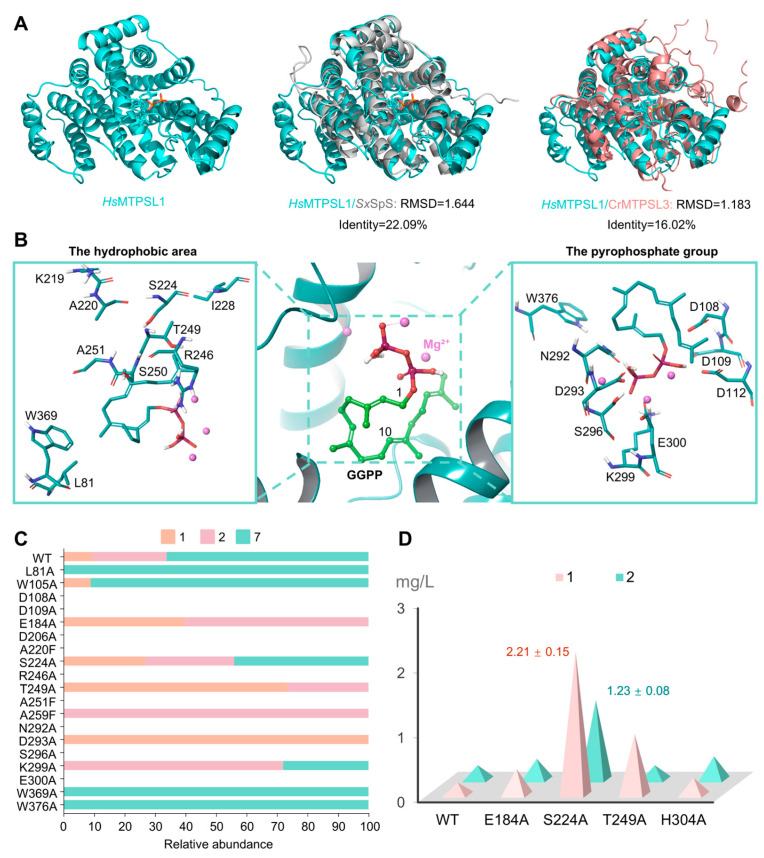
Mutational investigations of *Hs*MTPSL1. (**A**) Structural alignment of *Hs*MTPSL1 with *Sx*SpS and CrMTPSL3 predicted using AlphaFold3. (**B**) Docking model of *Hs*MTPSL1 with GGPP and three Mg^2+^ ions. GGPP is shown in stick representation (green carbons and red OPP), with the carbon atoms at positions 1 and 10 indicated, and Mg^2+^ ions are shown as pink spheres. Critical residues were shown. (**C**) The relative amounts of compounds **1**, **2**, and **7** produced by wild-type *Hs*MTPSL1 and its mutants. (**D**) Mutants with increased yield of compounds **1** and **2**. Yields were calculated through statistical analysis of three replicate experiments (mean ± SD) (Figure S25 and Table S2).

Due to the low abundance of compound **1**, we attempted to enhance its production by *Hs*MTPSL1 via protein engineering. To this end, we first predicted the structure of *Hs*MTPSL1 using AlphaFold3. Sequence and structural alignment of *Hs*MTPSL1 with CrMTPSL3 from the non-seed plant *Claopodium rostratum* and the bacterial spatane synthase *Sx*SpS revealed that, despite limited sequence similarity, their three-dimensional structures are highly conserved (Figure 3A and Figure S24 and Table S4). Structural alignment with the *Sx*SpS revealed critical residues (A220, S224, I228 in *Hs*MTPSL1; corresponding to C177, A181, A185 in *Sx*SpS) likely regulate access to the hydrophobic substrate-binding pocket. Molecular docking simulations with three Mg^2+^ ions and GGPP further supported this model, showing GGPP in a pre-twisted conformation with a C1–C10 distance of 3.48 Å, consistent with a cyclization-competent pose. Key conserved motifs involved in substrate binding, including DDXXD (residues 108–112) and NDXXSXXXE (residues 292–300), were identified along with other proximal residues (Figure 3B).

Systematic site-directed mutagenesis targeting these candidate residues was performed. Mutations at D108A, D109A, D206A, A220F, R246A, A251F, N292A, S296A and E300A resulted in the loss of all seven detected products, underscoring their essential role in enzymatic activity (Figure S21). In addition, we observed that beyond these residues, the L81A mutation led *Hs*MTPSL1 to produce only compound **7**; the W105A mutation abolished the production of both compounds **2** and **3**; the E184A and T249A mutations eliminated the production of the compound **7**; the D293A mutation resulted in the exclusive production of compound **1**; the K299A mutation blocked the formation of compound **1** while allowing all other compounds to be produced; the W369A mutation abolished compounds **1** and **2**; and the W376A mutation eliminated compounds **1**, **2**, and **3**. Encouragingly, mutations at E184A, S224A, T249A, and H304A enhanced the yields of compounds **1** and **2** (Figure 3C and Figure S21). Notably, the S224A mutation increased the production of compound **1** by 11.6-fold to 2.21 mg/L and compound **2** by 6-fold to 1.23 mg/L, while also elevating the yields of all other diterpenes (Figure 3D and Figure S25 and Table S2). These results indicate that, in addition to mutations in conserved substrate-binding motifs that affect overall activity of *Hs*MTPSL1, the enzyme has evolved unique residues that specifically abolish individual products within its multi-product profile.

### 2.3. Evolutionary Conservation Analysis of HsMTPSL1

**Figure 4:**
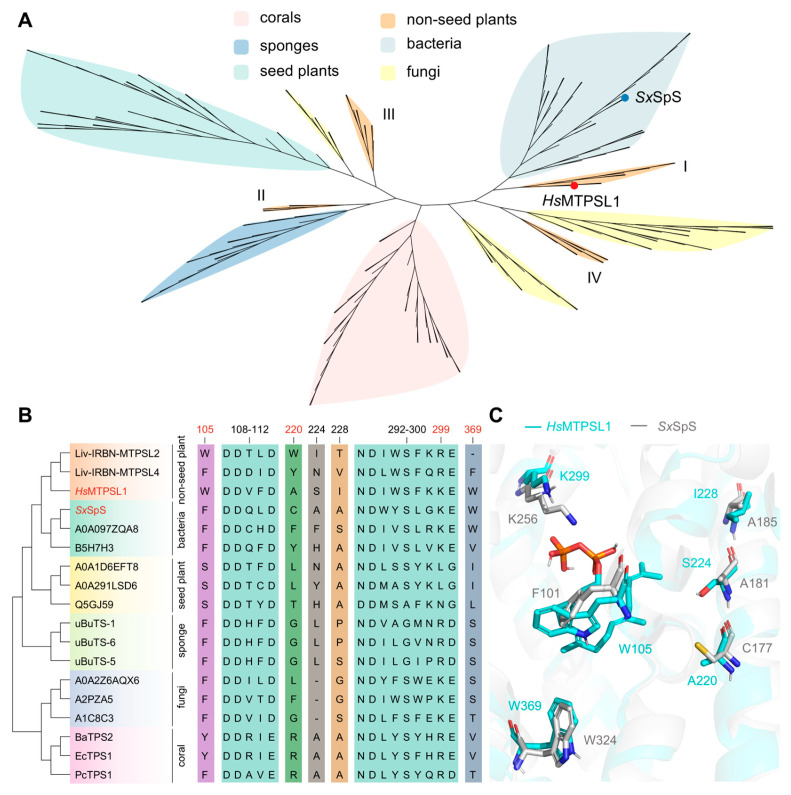
Evolutionary conservation analysis of *Hs*MTPSL1. (**A**) Phylogenetic analysis of *Hs*MTPSL1 with TPSs from corals, sponges, bacteria, fungi, non-seed plants, and seed plants. I/II/III/IV indicate the four distinct clades (Groups I/II/III/IV) in non-seed plants, respectively [9]. (**B**) Multiple sequence alignment of residues governing *Hs*MTPSL1 activity across terpene synthases from diverse lineages. (**C**) Alignment of key amino acid sites in *Hs*MTPSL1 and *Sx*SpS. The cyan residues belong to *Hs*MTPSL1, and the gray residues belong to *Sx*SPS.

To investigate the conservation and divergence of the key functional residues identified in *Hs*MTPSL1 between marine and terrestrial terpene synthases, we performed a phylogenetic analysis of terpene synthases across marine and terrestrial organisms, including corals, sponges, bacteria, fungi, non-seed plants, and seed plants, revealing that *Hs*MTPSL1 is evolutionarily more closely related to bacterial enzymes (Figure 4A). Multiple sequence alignment revealed that the core substrate-binding motifs DDXXD (residues 108–112) and NDXXSXXXE (residues 292–300), including K299 (essential for the production of **1** but not **2**), are highly conserved between *Hs*MTPSL1 and other TPSs. Notably, however, several functionally determinant residues identified in our mutagenesis study exhibited clear divergence. For instance, W105 (required for **2** but dispensable for **1**) and S224 (which dramatically enhanced yields of both compounds upon mutation) were not conserved across the aligned sequences (Figure 4B). While W369 (required for **1** and **2**) is identical in *Hs*MTPSL1 and *Sx*SpS, it is divergent in terpene synthases of marine animal and seed plant origins (Figure 4B,C). The three amino acid residues that shape the hydrophobic substrate-binding channel (all of which influence the product profile) also differ between *Hs*MTPSL1 and *Sx*SpS (Figure 4C). These findings suggest that while the lycophyte enzyme *Hs*MTPSL1 retains fundamental catalytic motifs shared with its marine bacterial counterparts, it has also evolved distinct amino acid residues that fine-tune product specificity and efficiency, likely enabling the formation of a unique diterpenoid profile within the plant host. This reflects a functional divergence following the evolutionary separation of terrestrial and marine biosynthetic pathways.

**Figure 5:**
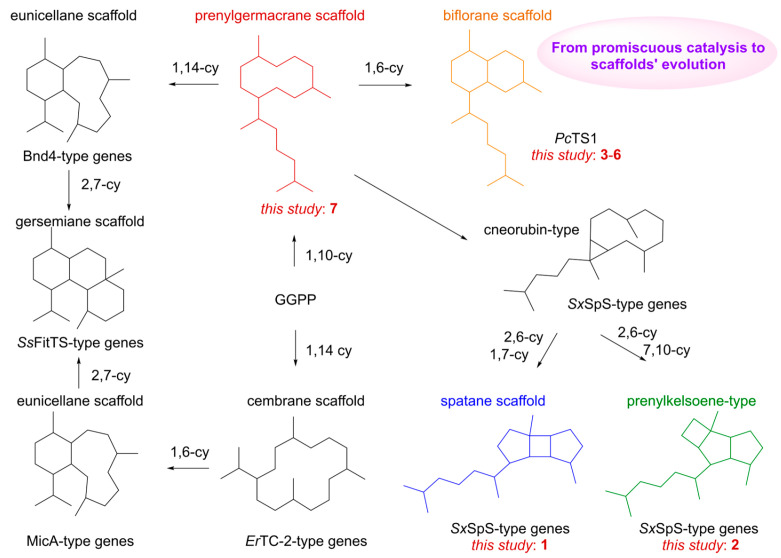
Hypothesis for terpene scaffold evolution centered on the ancient terpene synthase. The 1,10-cyclization of GGPP generates the prenylgermacrane scaffold, a central 10-membered macrocyclic intermediate. From this hub, 1,6-cyclization leads to the biflorane skeleton [15], whereas 1,14-cyclization leads to the eunicellane skeleton [30,31], which can further undergo 2,7-cyclization to form the gersemiane skeleton [32,33]. Alternatively, the prenylgermacrane scaffold can be converted into cneorubin-type intermediates [17]; subsequent 2,6- and 1,7-cyclizations produce the spatane scaffold [17], while 2,6- and 7,10-cyclizations yield prenylkelsoene-type compounds [17]. In addition, direct 1,14-cyclization of GGPP forms the cembrane skeleton [34], followed by 1,6-cyclization to eunicellane [35] and then 2,7-cyclization to gersemiane [32].

The distinct product profiles of marine versus terrestrial terpene synthases are largely dictated by their sequence divergence. The pronounced product promiscuity of *Hs*MTPSL1 prompted us to investigate the systematic diversification of diterpene scaffolds originating from terpene synthase. Analysis of *Hs*MTPSL1’s products points to a key 10-membered macrocyclic intermediate: the prenylgermacrane scaffold. This is formed via 1,10-cyclization of GGPP. From this central scaffold, 1,6-cyclization leads to the biflorane skeleton [15], while 1,14-cyclization yields the eunicellane skeleton [30,31]. The latter can further undergo 2,7-cyclization to generate the gersemiane skeleton [32,33]. Furthermore, the prenylgermacrane scaffold can be transformed into cneorubin-type compounds [17], which subsequently undergo two distinct cyclization routes: 2,6- and 1,7-cyclizations produce the spatane scaffold, whereas 2,6- and 7,10-cyclizations give rise to prenylkelsoene-type compounds [17]. Beyond the 1,10-cyclization route, GGPP can also undergo 1,14-cyclization to form the cembrane skeleton [34], which subsequently undergoes 1,6-cyclization to yield the eunicellane scaffold [35], followed again by 2,7-cyclization to form the gersemiane skeleton [32] (Figure 5). This interconnected network of skeletal transformations highlights an underlying biosynthetic link between marine diterpenoid systems and ancient terrestrial plants. Ultimately, the connectivity of these scaffolds stems from the relatedness of the enzyme sequences themselves, which in turn traces back to the shared evolutionary history of the organisms that harbor them.

### 2.4. Expression Profile and Co-Expression Analysis of CYP450s with HsMTPSL1

**Figure 6:**
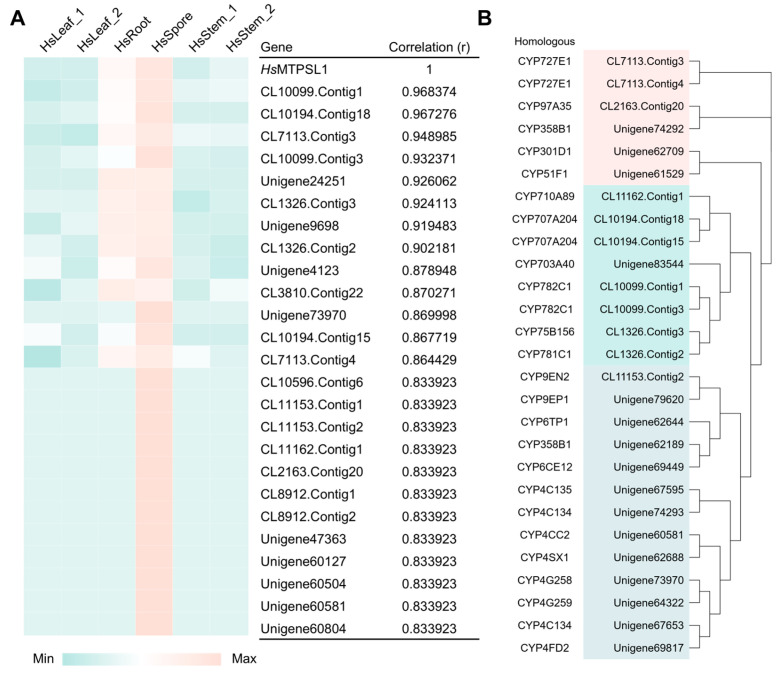
Co-expression analysis of *Hs*MTPSL1 and CYP450s. (**A**) Co-expression analysis of *Hs*MTPSL1 with CYP450s in different tissues of *H. serrata.* (**B**) Phylogenetic and homology analysis of highly co-expressed P450s.

Diterpene scaffolds are typically subjected to oxidative modifications by cytochrome P450 enzymes (CYPs) to generate bioactive derivatives. To identify candidate CYPs potentially involved in tailoring the diterpene products of *Hs*MTPSL1, we first analyzed the expression profile of *Hs*MTPSL1 across different tissues of *H*. *serrata* [14]. Transcriptomic data revealed that *Hs*MTPSL1 is predominantly expressed in sporangia (Figure 6A). We then performed co-expression analysis between *Hs*MTPSL1 and all annotated CYP450 genes in the transcriptome. Several CYPs showed a strong correlation with *Hs*MTPSL1 expression (Figure 6A). Phylogenetic analysis of these candidate CYPs, following homology searches via the P450 Atlas database, revealed distinct evolutionary branches. One branch comprised animal-like CYPs, while two other closely related clusters were of plant origin, primarily belonging to the CYP782, CYP707, CYP727, CYP75, and CYP781 families (Figure 6B and Table S5). Among these, CYP782 and CYP727 have been implicated in the biosynthesis of huperzine A [14]; CYP707 is involved in abscisic acid catabolism [36]; and CYP75 participates in flavonoid biosynthesis [37]. Notably, the function of CYP781 remains uncharacterized, suggesting it may represent a novel branch of P450s potentially involved in terpenoid modification (Figure 6). These co-expressed CYP450s represent promising candidates for the downstream oxidative diversification of the spatane, prenylkelsoene and biflorane diterpene scaffold.

## 3. Discussion

In this study, we report the discovery and functional characterization of *Hs*MTPSL1, a microbial-like terpene synthase from the lycophyte *Huperzia serrata*, representing the first plant-derived enzyme capable of generating the marine-associated spatane diterpene skeleton. Through heterologous expression and extensive product analysis, we demonstrated that *Hs*MTPSL1 is a multifunctional diterpene synthase that produces seven distinct diterpenes spanning three skeletal types—spatane, prenylkelsoene-type, and biflorane—with compound **7** identified as a novel biflorane diterpene. Structure-guided mutagenesis enabled the identification of key residues governing product specificity, including W105A, K299A, and W369A, which selectively control the formation of individual skeletons. Remarkably, the S224A mutation significantly enhanced the production of spatane diterpenes by up to 11.6-fold, demonstrating the potential for protein engineering to improve the yield of otherwise minor products.

Phylogenetic analysis placed *Hs*MTPSL1 in closer evolutionary proximity to bacterial terpene synthases than to higher plant TPSs, while sequence and structural alignments revealed both conserved catalytic motifs and lineage-specific residues that fine-tune product output, reflecting functional divergence following the separation of marine and terrestrial biosynthetic pathways. Furthermore, transcriptomic profiling showed that *Hs*MTPSL1 is highly expressed in sporangia and co-expressed with several cytochrome P450s, particularly those of the CYP781 family, implicating them as candidate tailoring enzymes for downstream oxidative modifications.

Future efforts, including using targeted metabolomics to determine whether the products of *Hs*MTPSL1 are present in plant tissues, genome mining of additional terpene synthases from early-diverging land plants and functional characterization of the candidate P450s identified herein, will further unravel the evolutionary and biochemical connections between marine and terrestrial terpene metabolism, and may enable the heterologous production of bioactive spatane diterpenes for pharmaceutical and biotechnological applications.

## 4. Materials and Methods

### 4.1. Instruments and Materials

Optical rotation data were acquired using a PerkinElmer 241 MC polarimeter (PerkinElmer, Fremont, CA, USA). NMR experiments were conducted at 298 K on Bruker Avance 600 MHz instruments (Bruker Biospin AG, Fallanden, Germany). Chemical shifts (*δ*) are reported in ppm relative to residual solvent signals (for CDCl_3_: *δ*_H_ 7.26, *δ*_C_ 77.16; for C_6_D_6_: *δ*_H_ 7.16, *δ*_C_ 128.06), and coupling constants (*J*) are given in Hz. Structural elucidation was assisted by 2D NMR techniques including ^1^H–^1^H COSY, HSQC, HMBC, and NOESY. Low- and high-resolution ESI mass spectra were recorded on a Bruker Esquire 3000 plus (Bruker Daltonics K. K., Kanagawa, Japan) and a Waters Q-TOF Ultima mass spectrometer (Waters, Milford, MA, USA), respectively. HPLC analysis was carried out on an Agilent 1260 system with a DAD G1315D detector (Agilent Technologies, Santa Clara, CA, USA) using a SB-C18 (150 mm × 4.6 mm, 5 µm) and a gradient of CH_3_CN–H_2_O at a flow rate of 1 mL/min. Column chromatography was performed with commercial silica gel (200–300 mesh). Analytical TLC used pre-coated HSGF-254 plates, and compounds were visualized under UV light or by staining with anisaldehyde–H_2_SO_4_ reagent followed by heating. All solvents were of analytical grade.

### 4.2. Isolation of Compounds

The codon-optimized sequence of *Hs*MTPSL1 was synthesized commercially (Universe Gene Technology (Tianjin) Co., Ltd., Tianjin, China). The N-terminal 20 amino acids of *Hs*MTPSL1 were truncated, and the coding sequence of the CC-Di-A acidic short peptide [16], followed by a GS linker, was fused to its N-terminus via overlap extension PCR. The resulting CC-Di-A-linker-trHsMTPSL1 fragment was then inserted into the pET28a vector using the *Nco*I and *Hind*III restriction sites. Mutants of *Hs*MTPSL1 were then amplified using this fusion gene as the template, with primers listed in Table S6. The resulting fragments were subsequently inserted into the pET28a vector using the *Bam*HI and *Hind*III restriction sites. These recombinant plasmids were then co-transformed separately with pCDF-MKI4 (Table S7) into *E. coli* BL21 Gold (DE3) (Weidibio, Shanghai, China) competent cells. Transformants were selected on lysogeny broth (LB) agar plates supplemented with kanamycin (50 mg/L) and streptomycin (50 mg/L). A single colony was inoculated into LB liquid medium containing the same antibiotics and grown overnight. The preculture was used to inoculate 30 L of fresh LB medium supplemented with 2.5% glycerol (1 L per flask). When the OD_600_ reached 2.0, expression was induced by adding isopropyl β-d-1-thiogalactopyranoside (IPTG, 0.5 mM) along with isoprenol (1.0 mM). Incubation continued at 28 °C with shaking at 220 rpm for 16 h. Cells were harvested by centrifugation (8000 *g*, 5 min), and the pellet was extracted with methanol. The methanolic extract was then partitioned with petroleum ether. The petroleum ether phase was concentrated under reduced pressure to give a yellowish oil (3.4 g). This crude material was fractionated by silica-gel column chromatography (200–300 mesh) and further purified by reversed-phase HPLC to yield compounds **3**–**7**.

For purification of the isomeric mixture of compounds **1** and **2**, silver nitrate (2.83 g) was ground to a fine powder and dissolved in methanol under stirring to form a homogeneous paste. Silica gel powder (10 g) was then added to the AgNO_3_ paste and mixed thoroughly by magnetic stirring for 10 min. The mixture was centrifuged to remove the solvent, and the resulting solid was sequentially washed with dichloromethane and petroleum ether (azeotropic mixture) to eliminate residual moisture. After rotary evaporation for 30 min, the silica gel-supported AgNO_3_ material was obtained as a dry powder. The prepared adsorbent was packed into a chromatography column. Prior to sample loading, the column was preconditioned by flushing with dichloromethane to remove traces of methanol and water, followed by rinsing with one column volume of 5% triethylamine in petroleum ether and three column volumes of pure petroleum ether. The crude mixture containing compounds **1** and **2** (2.89 mg) was then loaded onto the column. Initial elution with petroleum ether/ethyl acetate (100:1, *v*/*v*) yielded compound **2** as the first fraction, as monitored by TLC. Subsequent elution with petroleum ether/ethyl acetate (20:1, *v*/*v*) afforded compound **1**. After purification, 0.85 mg of compound **1** and 0.59 mg of compound **2** were obtained.

### 4.3. GC-MS Analysis

GC-MS analyses were performed on an Agilent 8890 gas chromatograph coupled to an Agilent 5977C mass spectrometer. The GC was equipped with an HP-5 ms capillary column (30 m × 0.25 mm, 0.25 μm film thickness). Samples (1 μL) were injected in split mode (3:1) with an inlet temperature of 250 °C. Helium carrier gas was maintained at a constant flow rate of 1.0 mL/min. The oven temperature program was as follows: 50 °C held for 2 min, increased at 10 °C/min to 250 °C, and held for 5 min, resulting in a total run time of 27 min. The MS was operated in electron ionization (EI) mode at 70 eV, with ion source, quadrupole, and transfer line temperatures set at 250 °C, 130 °C, and 250 °C, respectively. Full-scan data were collected over the range *m*/*z* 30–550. Compound identification was achieved by comparing mass spectra with the NIST 23 database using Agilent MassHunter software (v 10.2).

## 5. Conclusions

In summary, our findings establish *Hs*MTPSL1 as the first functionally characterized MTPSL from *H. serrata* and the first plant terpene synthase capable of producing spatane diterpenes. This work expands our understanding of terpenoid biosynthetic diversity in early-diverging plants, provides a molecular basis for engineering diterpene scaffold specificity, and offers a valuable enzyme platform for future synthetic biology applications aimed at producing complex marine-like diterpenes in heterologous hosts.

## Data Availability

The data presented in this study of *Hs*MTPSL1 are openly available at https://www.ncbi.nlm.nih.gov/genbank/ (accessed on 14 April 2026), reference number PX999718. The original contributions presented in this study are contained within the article or Supplementary Materials. Further inquiries can be directed to the corresponding authors.

## Supplementary Materials

The following supporting information can be downloaded at https://www.mdpi.com/article/10.3390/molecules31081329/s1, Compound structure elucidation; Figure S1: Comparison of EI mass spectra of compounds **1** and **2** with spata-13,17-diene and prenylkelsoene; Figure S2: ^1^H NMR spectrum of compound **1** in C_6_D_6_; Figure S3: ^13^C NMR spectrum of compound **1** in C_6_D_6_; Figure S4: ^1^H NMR spectrum of compound **2** in C_6_D_6_; Figure S5: ^13^C NMR spectrum of compound **2** in C_6_D_6_; Figure S6: ^1^H NMR spectrum of compound **3** in C_6_D_6_; Figure S7: ^13^C NMR spectrum of compound **3** in C_6_D_6_; Figure S8: ^1^H NMR spectrum of compound **4** in C_6_D_6_; Figure S9: ^13^C NMR spectrum of compound **4** in C_6_D_6_; Figure S10: ^1^H NMR spectrum of compound **5** in C_6_D_6_; Figure S11: ^13^C NMR spectrum of compound **5** in C_6_D_6_; Figure S12: ^1^H NMR spectrum of diepoxide-**6** in C_6_D_6_; Figure S13: ^13^C NMR spectrum of diepoxide-**6** in C_6_D_6_; Figure S14: ^1^H NMR spectrum of compound **7** in C_6_D_6_; Figure S15: ^13^C NMR spectrum of compound **7** in C_6_D_6_; Figure S16: ^1^H-^1^H COSY spectrum of compound **7** in C_6_D_6_; Figure S17: HSQC spectrum of compound **7** in C_6_D_6_; Figure S18: HMBC spectrum of compound **7** in C_6_D_6_; Figure S19: NOESY spectrum of compound **7** in C_6_D_6_; Figure S20: GC-MS spectrum of compound **7**; Figure S21: In vivo enzymatic activity comparison of wild-type *Hs*MTPSL1 and its mutants; Figure S22: Standard curve of compound **1**; Figure S23: Standard curve of compound **2**; Figure S24: Multiple sequence alignment of *Hs*MTPSL1 with *Sx*SpS and CrMTPSL3; Figure S25: Mutants with increased yield of compounds **1** and **2**; Table S1: ^1^H and ^13^C NMR data for **7** in C_6_D_6_; Table S2: The yield of compounds **1** and **2** of wild-type *Hs*MTPSL1 and its mutants; Table S3: MTPSLs from different species sources; Table S4: Protein sequence of *Hs*MTPSL1, *Sx*SpS and CrMTPSL3; Table S5: Highly co-expressed CYP450 genes in *Huperzia serrata* transcriptome and their homologous genes; Table S6: Primer sequences used in this study; Table S7: Plasmids used in this study.
