# Supplementary material for: Functional Characterization and Mutagenesis Studies of a Microbial-like Diterpene Synthase from Huperzia serrata
Source: Molecules. 2026 Apr 17;31(8):1329. doi: 10.3390/molecules31081329 (PMC13118282; doi:10.3390/molecules31081329)
Supplement: Supplementary file 1 [file molecules-31-01329-s001.zip › molecules-4255823-supplementary.pdf]

Supplementary materials

Functional characterization and mutagenesis studies of a microbial-like diterpene synthase from *Huperzia serrata*

Ting He [1,2,†], Yao Zhao [2,3,4,†], Xin Li [2,3], Bao Chen [2,3], Fangyan Chen [2,3,*] and Baofu Xu [2,3,*]

[1] Shandong University of Traditional Chinese Medicine, Jinan 250355, China.

[2] Shandong Laboratory of Yantai Drug Discovery, Bohai Rim Advanced Research Institute for Drug Discovery, Yantai, Shandong 264117, China.

[3] Shanghai Institute of Materia Medica, Chinese Academy of Sciences, Shanghai 201203, China.

[4] Nottingham Ningbo China Beacons of Excellence Research and Innovation Institute, University of Nottingham Ningbo, Ningbo, 315100, China.

* Correspondence: bfxu@simm.ac.cn (B.X.); or fychen@baridd.ac.cn (F.C.).

† These authors contributed equally to this work.

Table of contents

Compound structure elucidation **3**
Supplementary Figures **6**
Figure S1. Comparison of EI mass spectra of compounds **1** and **2** with spata-13,17-diene and prenylkelsoene. 6
Original experimental spectra of compound **1**. 7
Original experimental spectra of compound **2**. 8
Original experimental spectra of compound **3**. 9
Original experimental spectra of compound **4**. 10
Original experimental spectra of compound **5**. 11
Original experimental spectra of compound **6**. 12
Original experimental spectra of compound **7**. 13
Figure S20. GC-MS spectrum of compound **7** 16
Figure S21. In vivo enzymatic activity comparison of wild-type *Hs*MTPSL1 and its mutants. 17
Figure S22. Standard curve of compound **1**. 18
Figure S23. Standard curve of compound **2**. 18
Figure S24. Multiple sequence alignment of *Hs*MTPSL1 with *Sx*SpS and CrMTPSL3. 19
Figure S25. Mutants with increased yield of compounds **1** and **2** 20
Supplementary Tables **21**
Table S1. ^1H and ^{13}C NMR data for **7** in C_6D_6. 21
Table S2. The yield of compounds **1** and **2** of wild-type *Hs*MTPSL1 and its mutants. 22
Table S3. MTPSLs from different species sources. 23
Table S4. Protein sequence of *Hs*MTPSL1, *Sx*SpS and CrMTPSL3. 24
Table S5. Highly co-expressed CYP450 genes in *Huperzia serrata* transcriptome and their homologous genes. 25
Table S6. Primer sequences used in this study. 26
Table S7. Plasmids used in this study 29

Compound structure elucidation

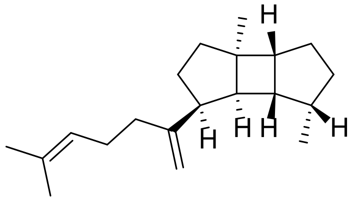

1

Compound **1** was identified as spata-13,17-diene. This structural assignment was supported by comparing its ^{1}H and ^{13}C NMR data (Figures S2 and S3) with literature values reported for the product derived from GGPP via *Sx*SpS in *Streptomyces xinghaiensis* [1].

Compound **1**: ^{1}H NMR (C_6D_6, 600 MHz) δ 5.27 (1H, m, vinyl H), 4.99 (1H, br s, vinyl H), 4.95 (1H, br s, vinyl H), 1.67 (3H, br s, CH_3), 1.55 (3H, br s, CH_3), 1.16 (3H, s, CH_3), 1.03 (3H, d, J = 7.0, CH_3); ^{13}C NMR ($CDCl_3$, 150 MHz) δ 148.88, 131.29, 125.00, 108.81, 49.17, 45.59, 44.29, 42.26, 42.01, 38.88, 37.29, 36.94, 35.06, 28.65, 28.50, 27.08, 25.86, 20.52, 17.75, 14.55.

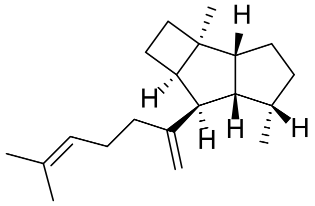

2

Compound **2** was characterized as prenylkelsoene. The identification was made by comparing its ^{1}H and ^{13}C NMR spectra (Figures S4 and S5) with previously reported data for the corresponding GGPP-derived product obtained via SxSpS in *S. Xinghaiensis* [1].

Compound **2**: ^{1}H NMR (C_6D_6, 600 MHz) δ 5.23 (1H, m, vinyl H), 5.05 (1H, br s, vinyl H), 4.95 (1H, br s, vinyl H), 1.67 (3H, br s, CH_3), 1.57 (3H, br s, CH_3), 0.93 (3H, s, CH_3), 0.90 (3H, d, J = 6.7, CH_3); ^{13}C NMR ($CDCl_3$, 150 MHz) δ 149.29, 131.17, 125.15, 109.73, 58.00, 50.18, 47.92, 47.10, 45.93, 37.92, 36.70, 33.64, 33.38, 26.97, 26.32, 25.86, 23.74, 18.17, 17.80, 15.16.

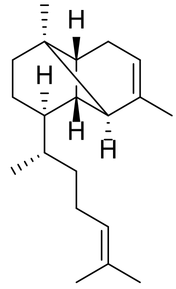

3

Compound **3** was obtained as a colorless oil and exhibited optical activity. Its ^{1}H NMR and ^{13}C NMR spectra (Figures S6 and S7) closely matched those reported for (+)-1,5-cyclo-5,8,9,10-tetrah ydroerogorgiaene, a known compound isolated from the brown alga *Dictyota dichotoma* [2], indi cating that both share the same relative configuration. However, the optical rotation of compou nd **3** {$[\alpha]^{20}_{D}$ –2.0 (c 0.20, $CHCl_3$)} was opposite in sign to that reported for (+)-1,5-cyclo-5,8,9,10-tetr ahydroerogorgiaene {$[\alpha]^{20}_{D}$ +4.0 (c 0.05, $CHCl_3$)}, clearly demonstrating that the two compounds a re enantiomers. Accordingly, compound **3** was identified as (–)-1,5-cyclo-5,8,9,10-tetrahydroero gorgiaene.

Compound **3**: ^{1}H NMR (C_6D_6, 600 MHz) δ 5.27 (1H, m, vinyl H), 5.23 (1H, m, vinyl H), 1.68 (3H, s, CH_3), 1.67 (3H, br s, CH_3), 1.58 (3H, s, CH_3), 0.90 (3H, s, CH_3), 0.85 (3H, d, J = 6.8, CH_3); ^{13}C NMR ($CDCl_3$, 150 MHz) δ 143.87, 130.90, 125.63, 116.68, 55.25, 45.70, 43.32, 39.62, 37.64, 37.37, 36.52, 34.64, 30.34, 26.36, 25.92, 23.29, 20.60, 19.50, 17.75, 15.67.

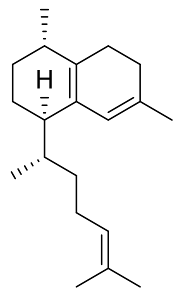

4

Compound **4** was identified as isoelisabethatriene. This assignment was based on comparison of its ^{1}H and ^{13}C NMR data (Figures S8 and S9) with those reported in the literature for the product of hydropyrene synthase incubated with GGPP [3].

Compound **4**: ^{1}H NMR (C_6D_6, 600 MHz) δ 5.79 (1H, s, vinyl H), 5.27 (1H, m, vinyl H), 1.76 (3H, s, CH_3), 1.71 (3H, br s, CH_3), 1.59 (3H, s, CH_3), 0.97 (3H, d, J = 6.9, CH_3), 0.85 (3H, d, J = 6.9, CH_3); ^{13}C NMR ($CDCl_3$, 150 MHz) δ 133.30, 132.32, 130.93, 130.31, 125.63, 122.75, 41.03, 35.73, 34.47, 34.04, 31.51, 29.05, 27.44, 26.87, 25.94, 23.28, 21.10, 19.22, 17.81, 15.04.

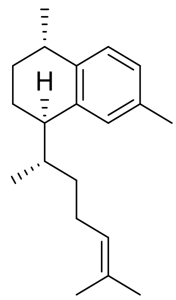

5

Compound **5** was identified as erogorgiaene by comparison of its ^{1}H and ^{13}C NMR spectroscopic data (Figures S10 and S11) with those reported in the literature [3,4], and further supported by its optical rotation of $[\alpha]_D^{20}$ +30.0 (*c* 0.18, $CHCl_3$).

Compound **5**: ^{1}H NMR (C_6D_6, 600 MHz) δ 7.16 (1H, d, J = 7.5, vinyl H), 7.11 (1H, br s, vinyl H), 6.96 (1H, d, J = 7.5, vinyl H), 5.28 (1H, m, vinyl H), 2.21 (3H, s, CH_3), 1.72 (3H, br s, CH_3), 1.61 (3H, s, CH_3), 1.23 (3H, d, J = 6.8, CH_3), 0.72 (3H, d, J = 6.8, CH_3); ^{13}C NMR ($CDCl_3$, 150 MHz) δ 140.62, 139.99, 134.88, 131.08, 128.51, 126.97, 126.58, 125.50, 41.96, 37.55, 35.66, 33.26, 32.21, 26.78, 25.95, 22.15, 22.01, 21.31, 17.81, 14.79.

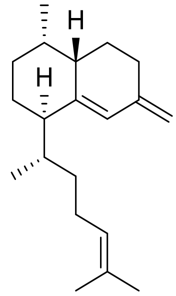

6

Compound **6** was identified as elisabethatriene through comparison of its ^{1}H and ^{13}C NMR spectroscopic data (Figures S12 and S13) with those reported in the literature [5].

Compound **6**: ^{1}H NMR (C_6D_6, 600 MHz) δ 6.14 (1H, s, vinyl H), 5.21 (1H, m, vinyl H), 4.87 (1H, s, vinyl H), 4.78 (1H, s, vinyl H), 1.65 (3H, s, CH_3), 1.59 (3H, br s, CH_3), 0.90 (3H, d, J = 6.5, CH_3), 0.86 (3H, d, J = 7.0, CH_3); ^{13}C NMR ($CDCl_3$, 150 MHz) δ 144.02, 143.94, 131.00, 127.55, 125.62, 109.01, 49.99, 37.12, 35.51, 35.21, 31.96, 29.85, 29.63, 27.53, 25.90, 25.89, 23.22, 17.80, 17.68, 15.19.

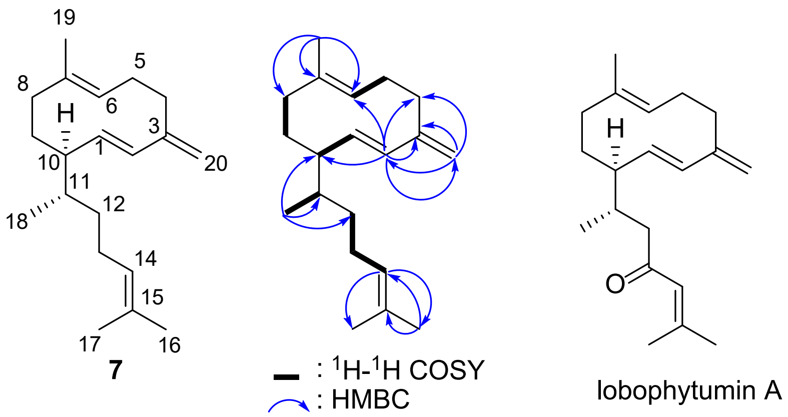

Compound **7** was isolated as a colorless oil. Its molecular formula was established as $C_{20}H_{32}$ based on the GC-MS ion peak observed at *m/z* 272.2 (Figure S20). Comparison of the ^{1}H and ^{13}C NMR data of **7** with those reported for lobophytumin A, a metabolite isolated from the soft coral *Lobophytum cristatum* Tixier-Durivault [6], revealed a high degree of structural similarity. The principal difference lay in the replacement of a carbonyl group in the known compound by a methylene unit in **7**, corresponding to a 14 Da reduction in molecular weight. This modification was supported by key HMBC correlations from (δ_H 0.92) to the C-12 (δ_C 34.0) and from Me-16 (δ_H 1.68) to C-14 (δ_C 125.6), as well as by ^{1}H–^{1}H COSY correlations between H-12 (δ_H 1.57, 1.13) and H-13 (δ_H 2.12), and between H-13 and H-14 (δ_H 5.23). Comprehensive analysis of 2D NMR data (HSQC, ^{1}H–^{1}H COSY, HMBC) confirmed that compound **7** is the C-13 decarbonylated analogue of lobophytumin A. The *E* configuration of the C1/C2 double bond was determined by a large coupling constant ($^{3}J_{H\text{-}1,\,H\text{-}2}$ = 15.8 Hz).The configuration of **7** was tentitively assigned based on the biosynthetic considerations.

Supplementary Figures

A B

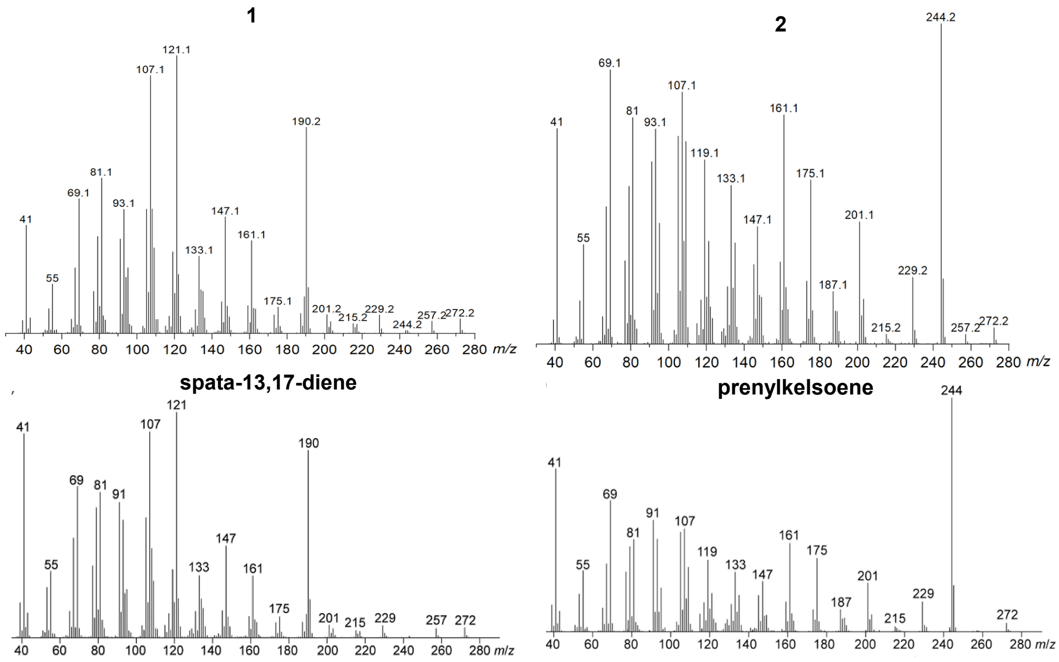

Figure S1. Comparison of EI mass spectra of compounds **1** and **2** with spata-13,17-diene and prenylkelsoene.(A) Comparison of EI mass spectra of compounds **1** and spata-13,17-diene [1]. (B) Comparison of EI mass spectra of compounds **2** and prenylkelsoene [1].

Original experimental spectra of compound **1**.

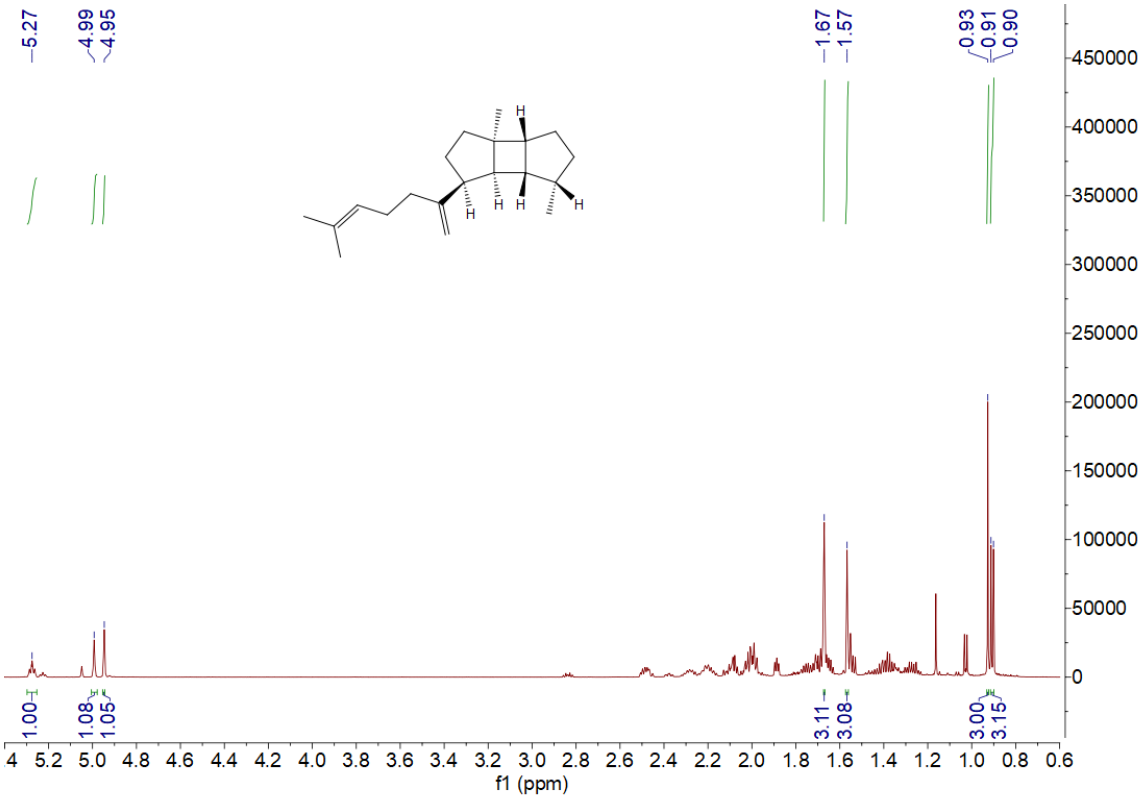

Figure S2. ^{1}H NMR spectrum of compound **1** in C_6D_6.

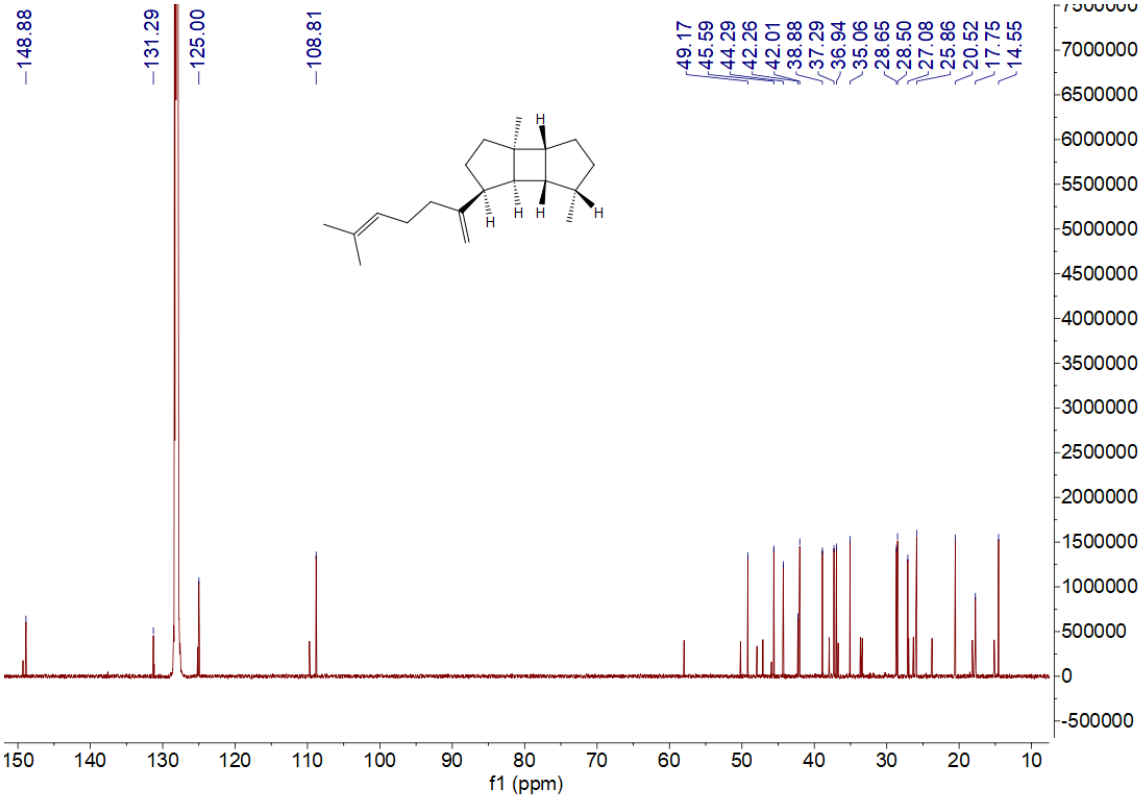

Figure S3. ^{13}C NMR spectrum of compound **1** in C_6D_6.

Original experimental spectra of compound **2**.

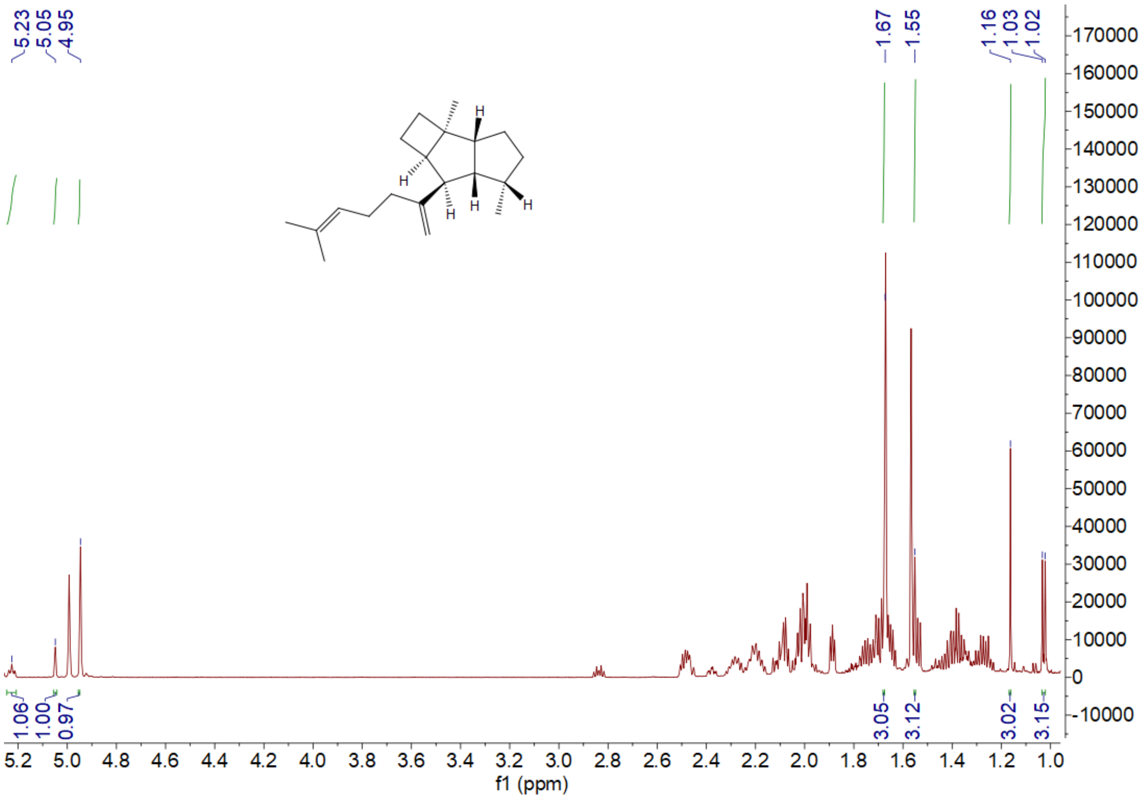

Figure S4. ^{1}H NMR spectrum of compound **2** in C_6D_6.

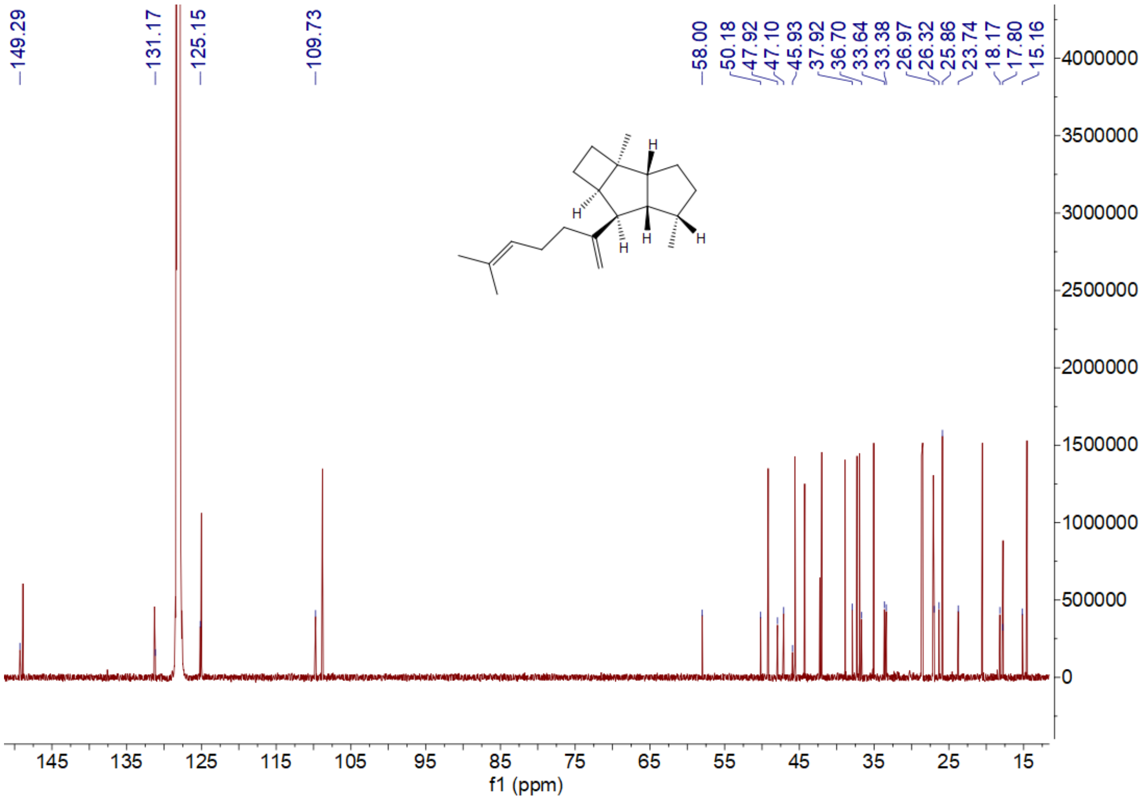

Figure S5. ^{13}C NMR spectrum of compound **2** in C_6D_6.

Original experimental spectra of compound **3**.

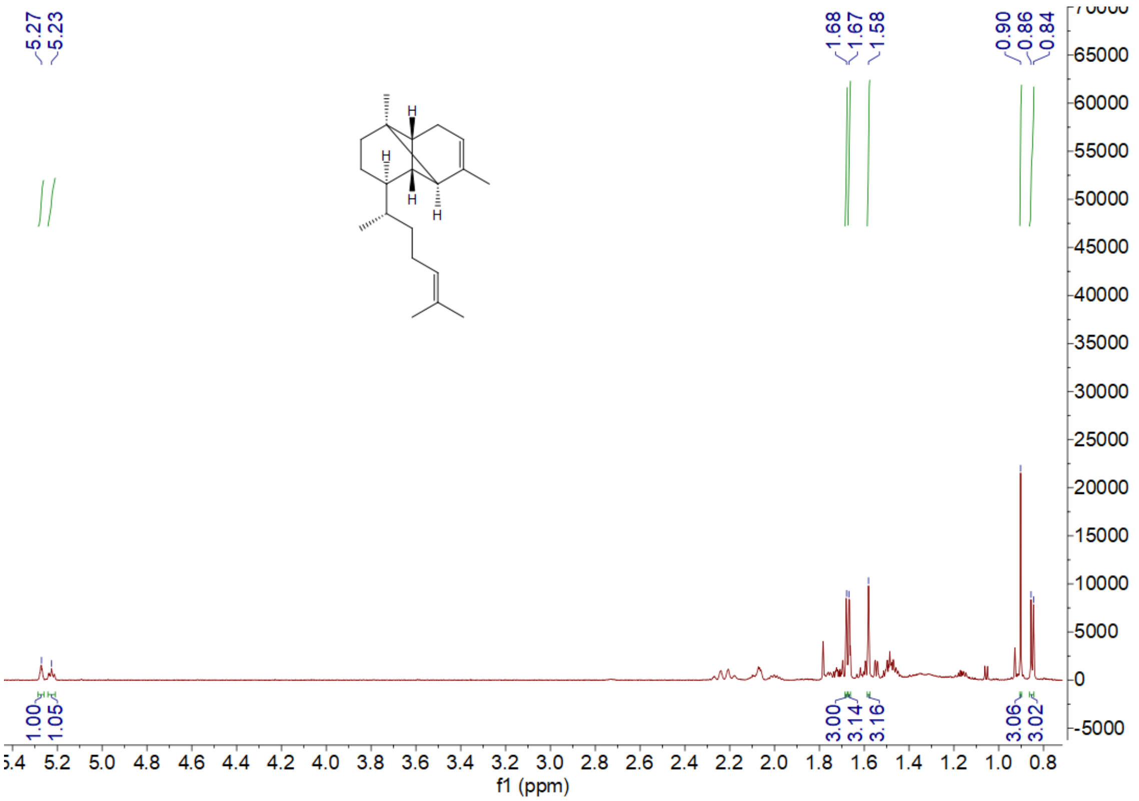

Figure S6. ^{1}H NMR spectrum of compound **3** in C_6D_6.

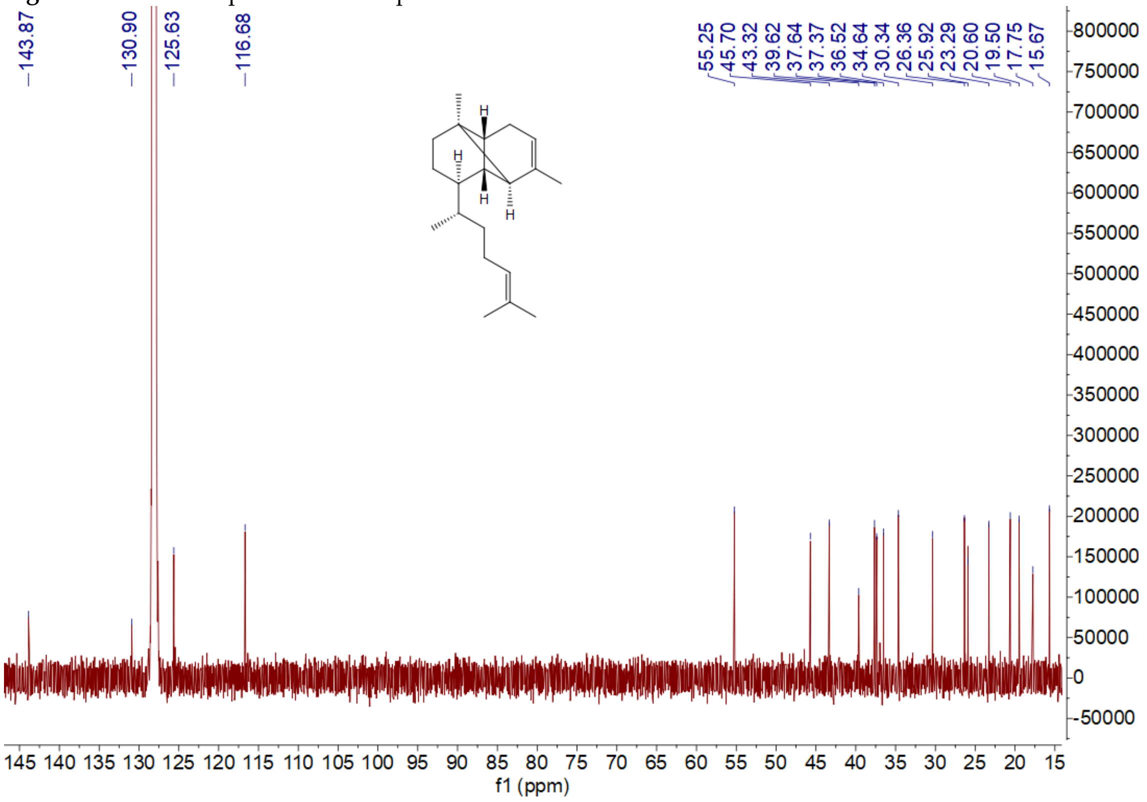

Figure S7. ^{13}C NMR spectrum of compound **3** in C_6D_6.

Original experimental spectra of compound **4**.

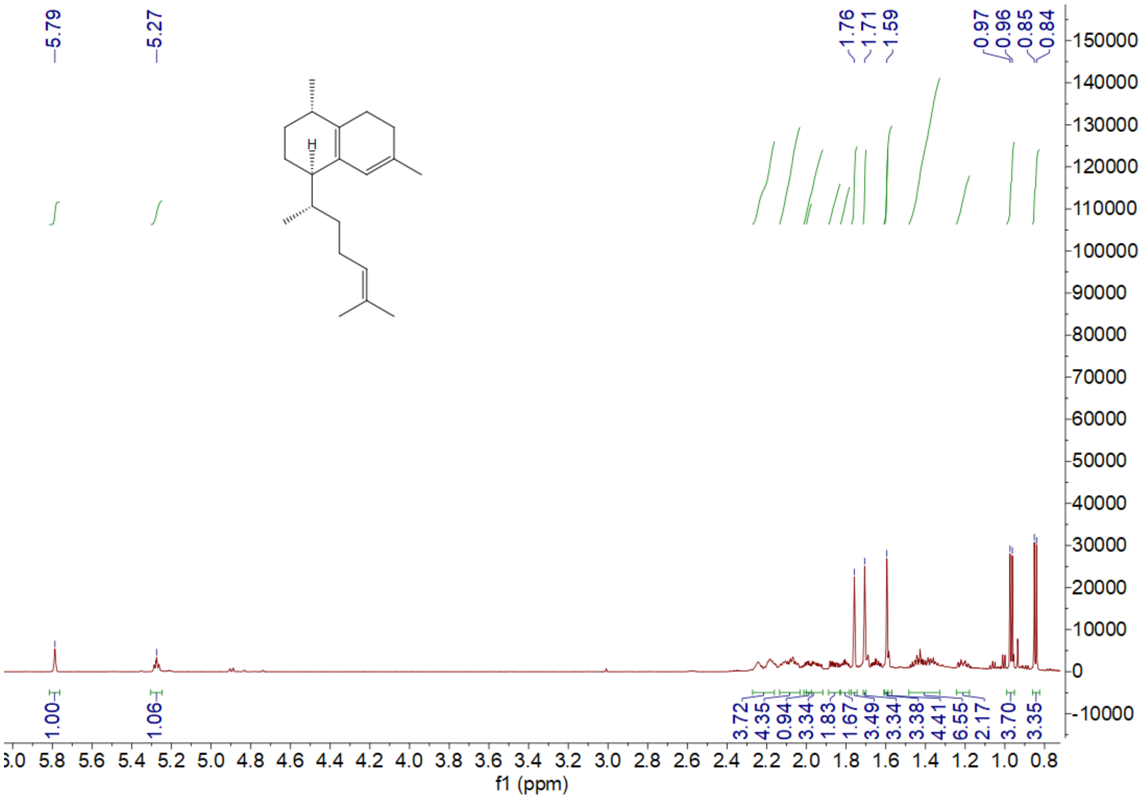

Figure S8. ^{1}H NMR spectrum of compound **4** in C_6D_6.

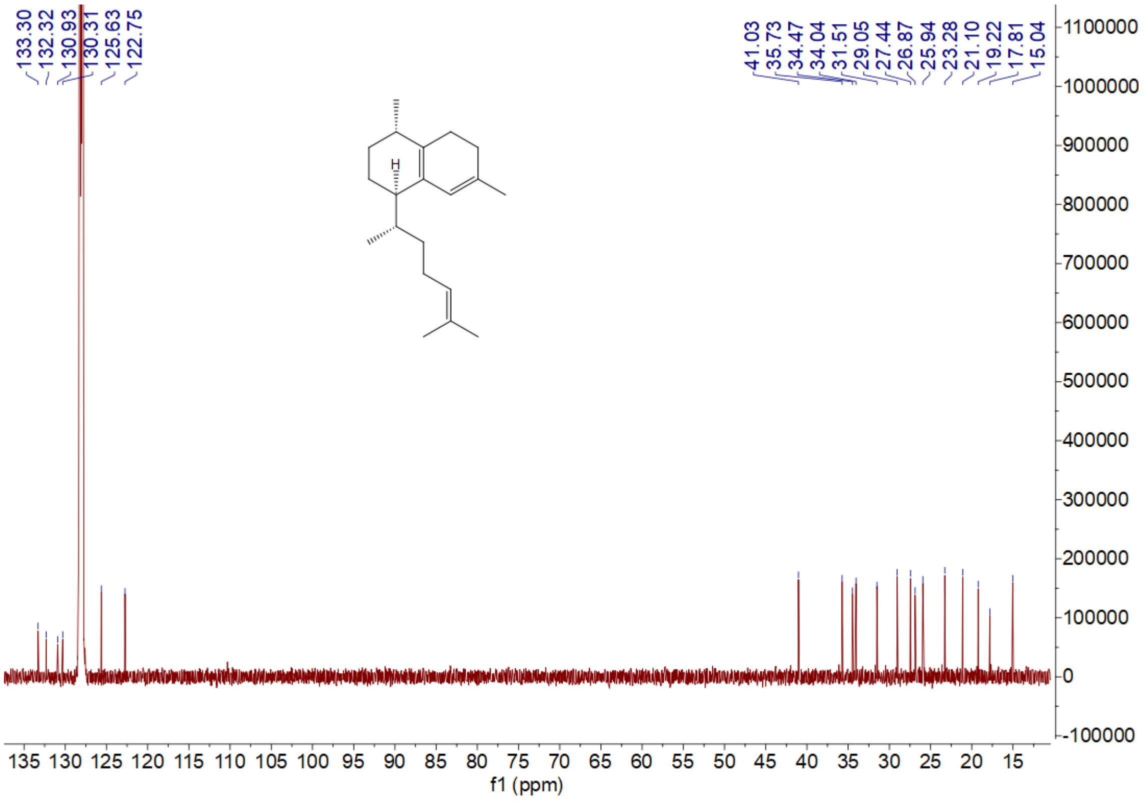

Figure S9. ^{13}C NMR spectrum of compound **4** in C_6D_6.

Original experimental spectra of compound **5**.

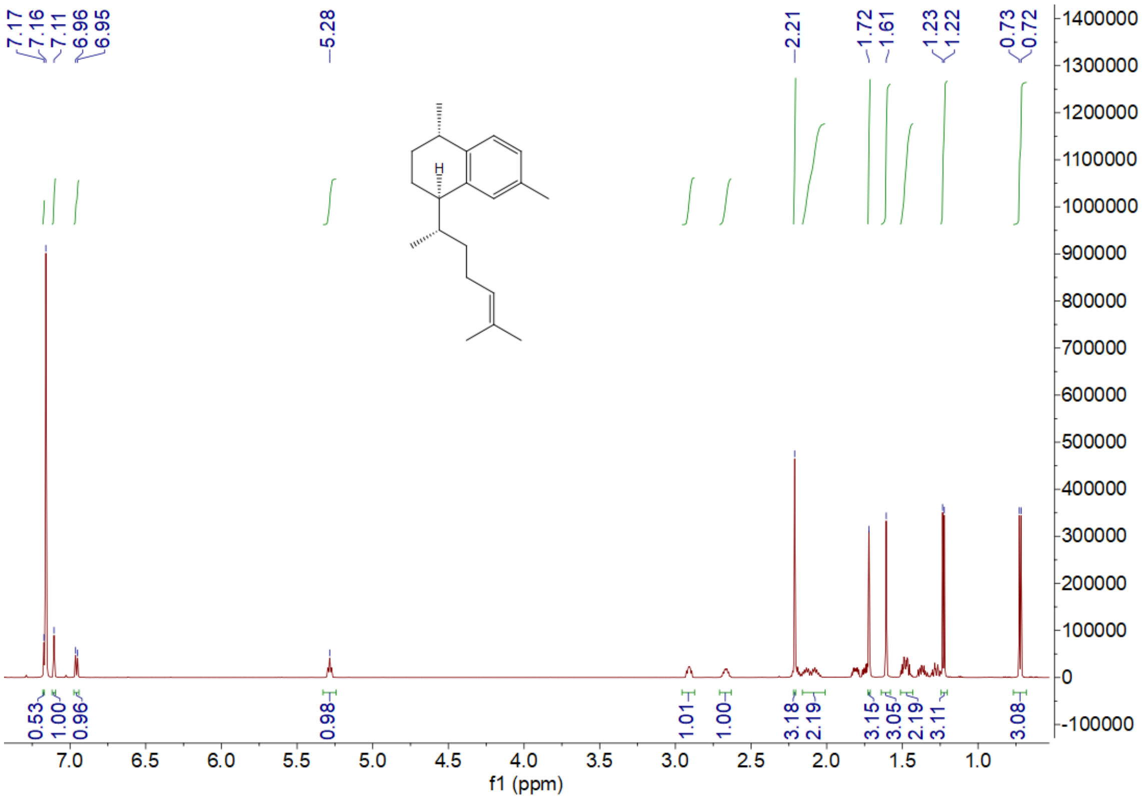

Figure S10. ^{1}H NMR spectrum of compound **5** in C_6D_6.

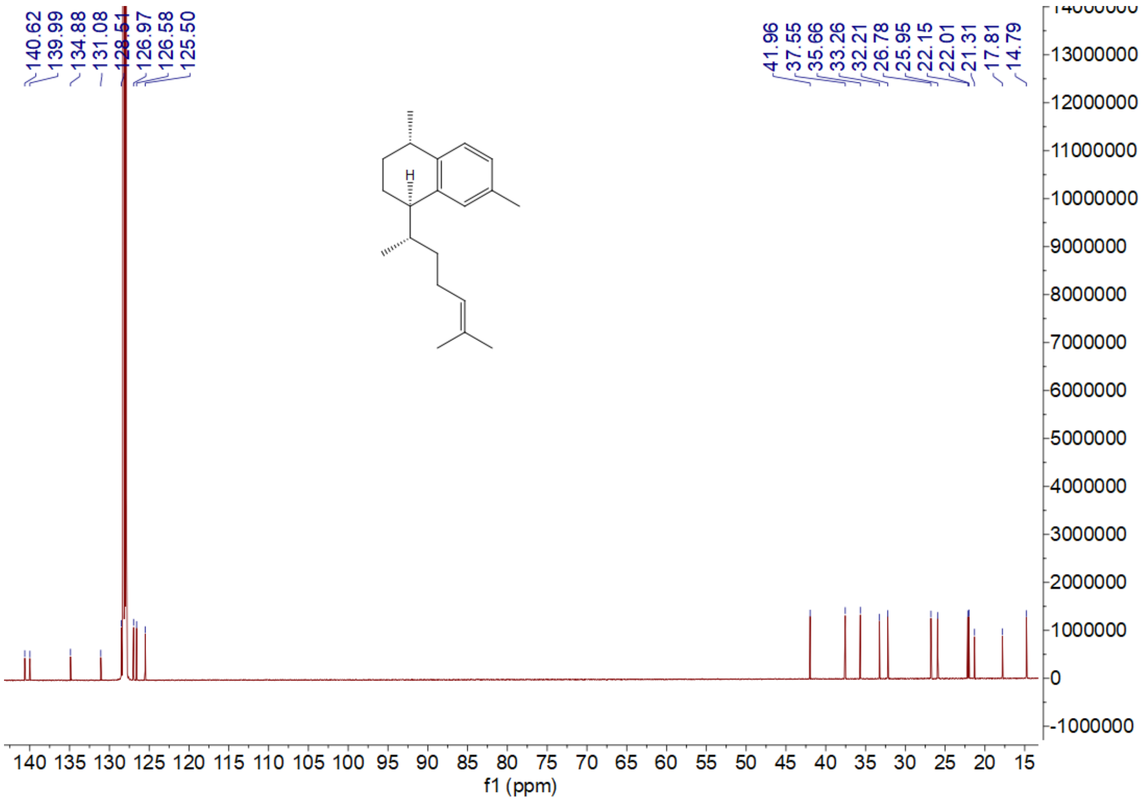

Figure S11. ^{13}C NMR spectrum of compound **5** in C_6D_6.

Original experimental spectra of compound **6**.

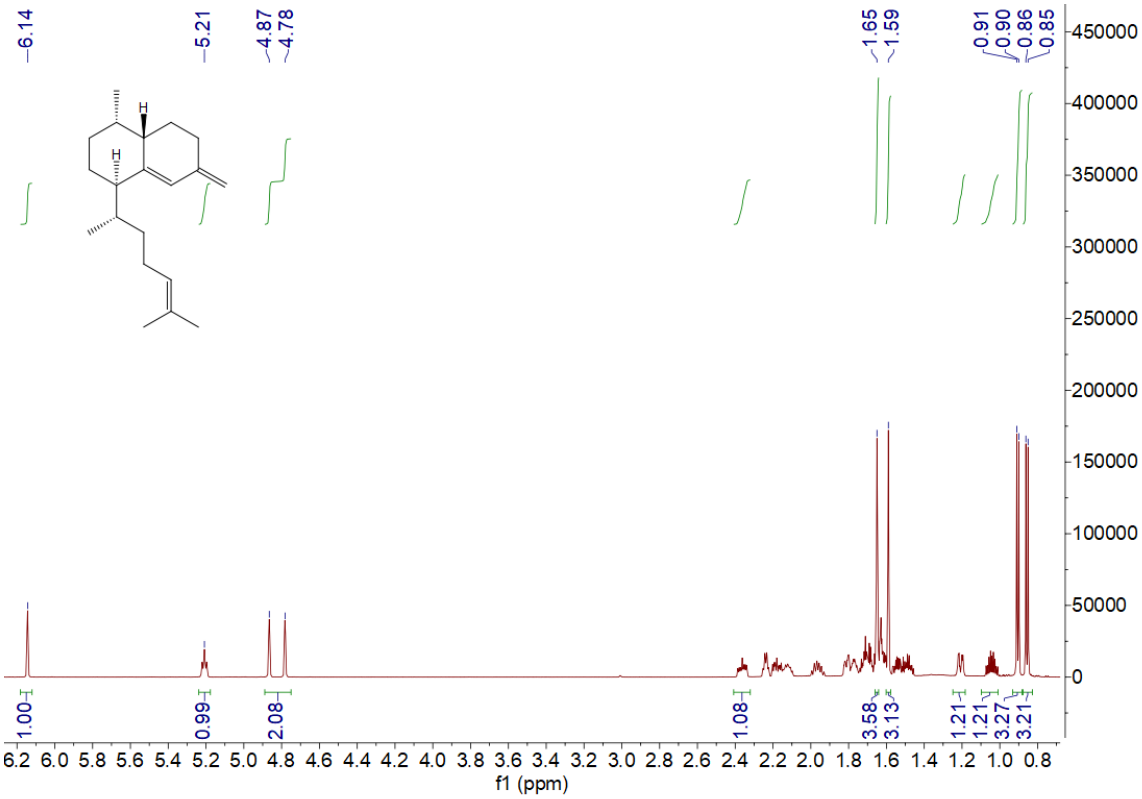

Figure S12. ^{1}H NMR spectrum of diepoxide-**6** in C_6D_6.

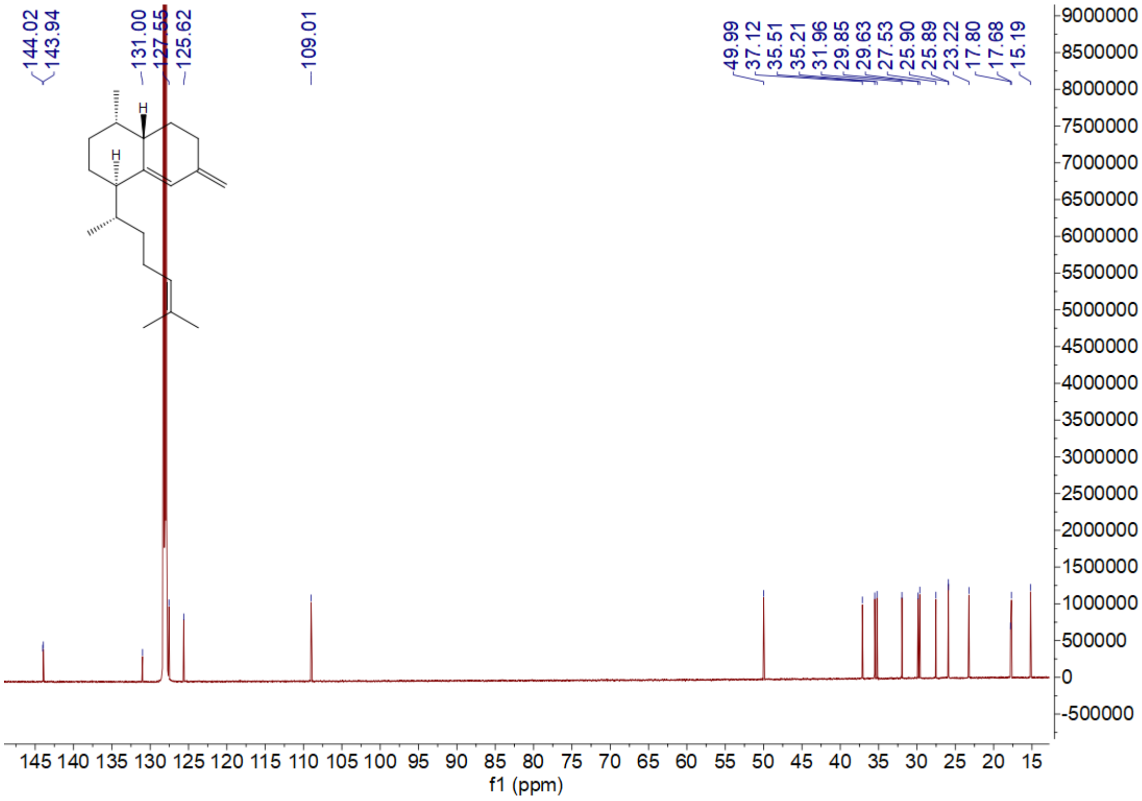

Figure S13. ^{13}C NMR spectrum of diepoxide-**6** in C_6D_6.

Original experimental spectra of compound **7**.

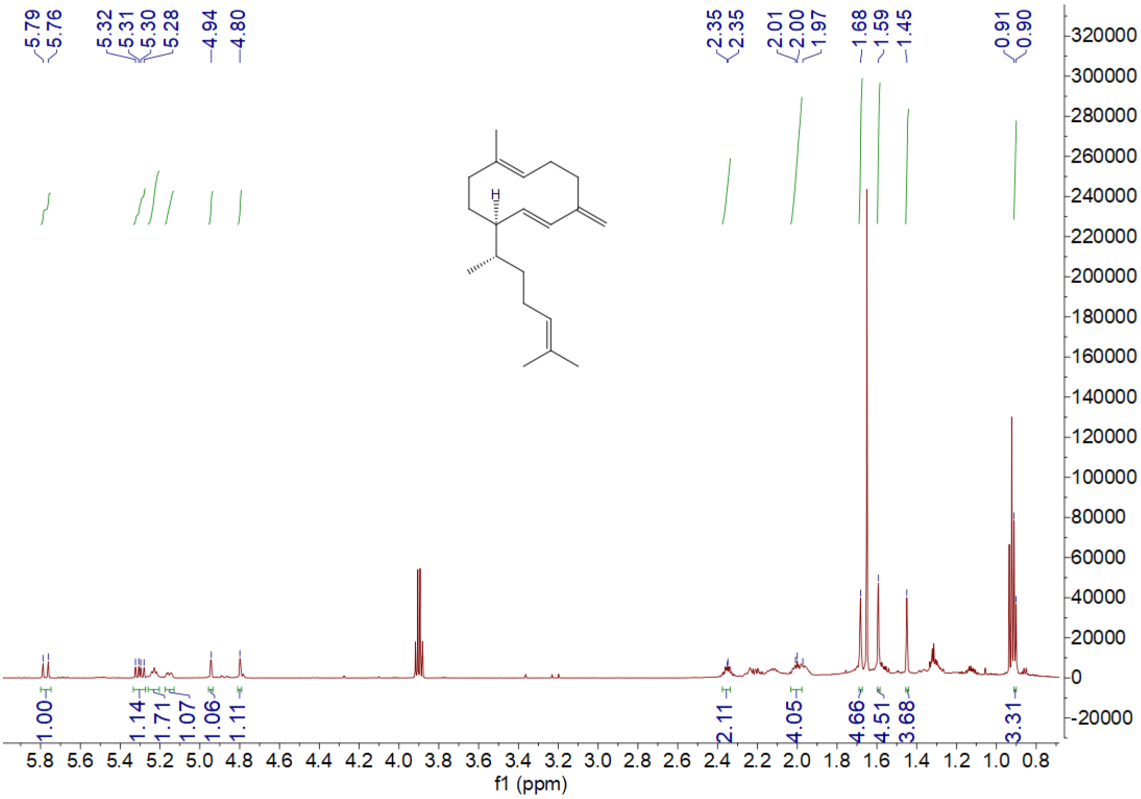

Figure S14. ^{1}H NMR spectrum of compound **7** in C_6D_6.

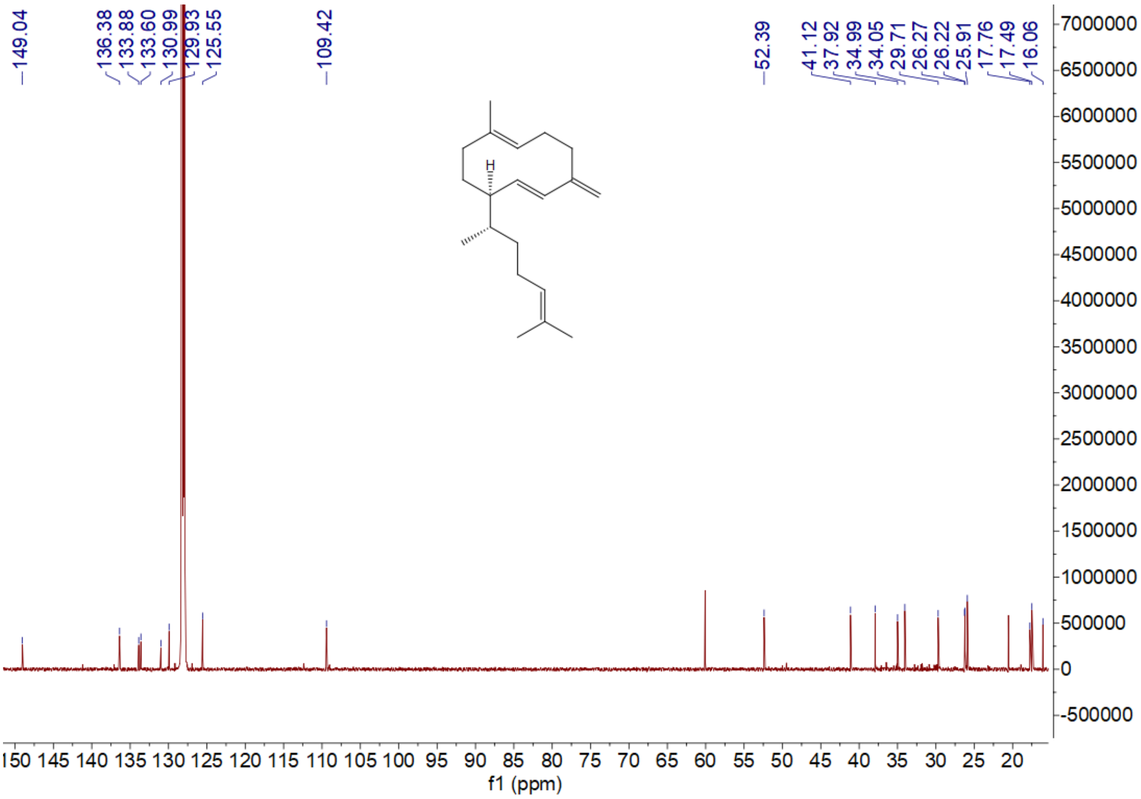

Figure S15. ^{13}C NMR spectrum of compound **7** in C_6D_6.

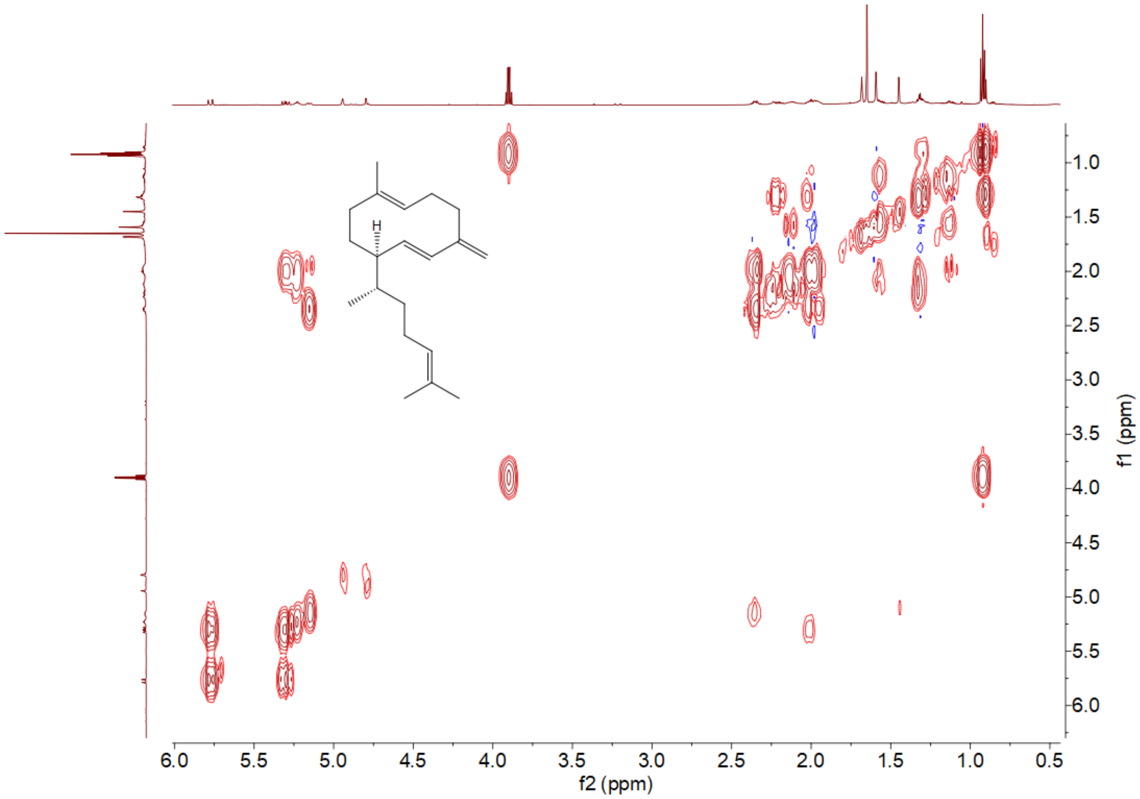

Figure S16. ^{1}H-^{1}H COSY spectrum of compound **7** in C_6D_6.

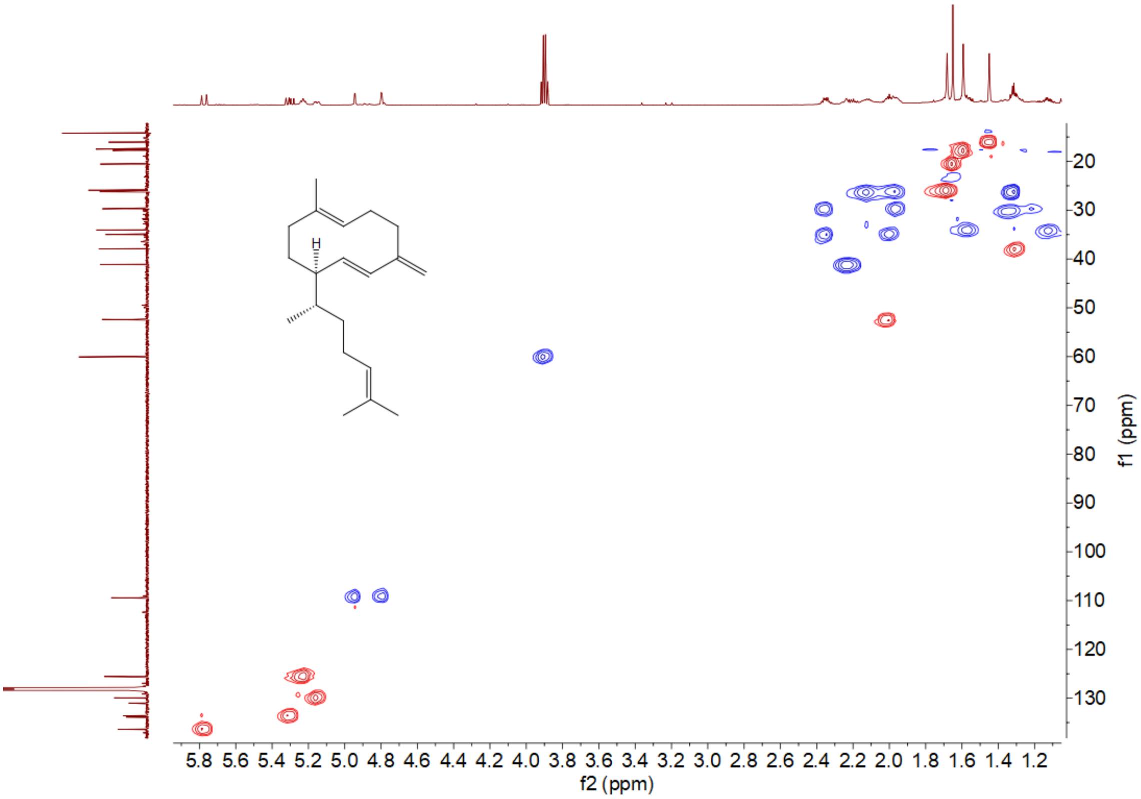

Figure S17. HSQC spectrum of compound **7** in C_6D_6.

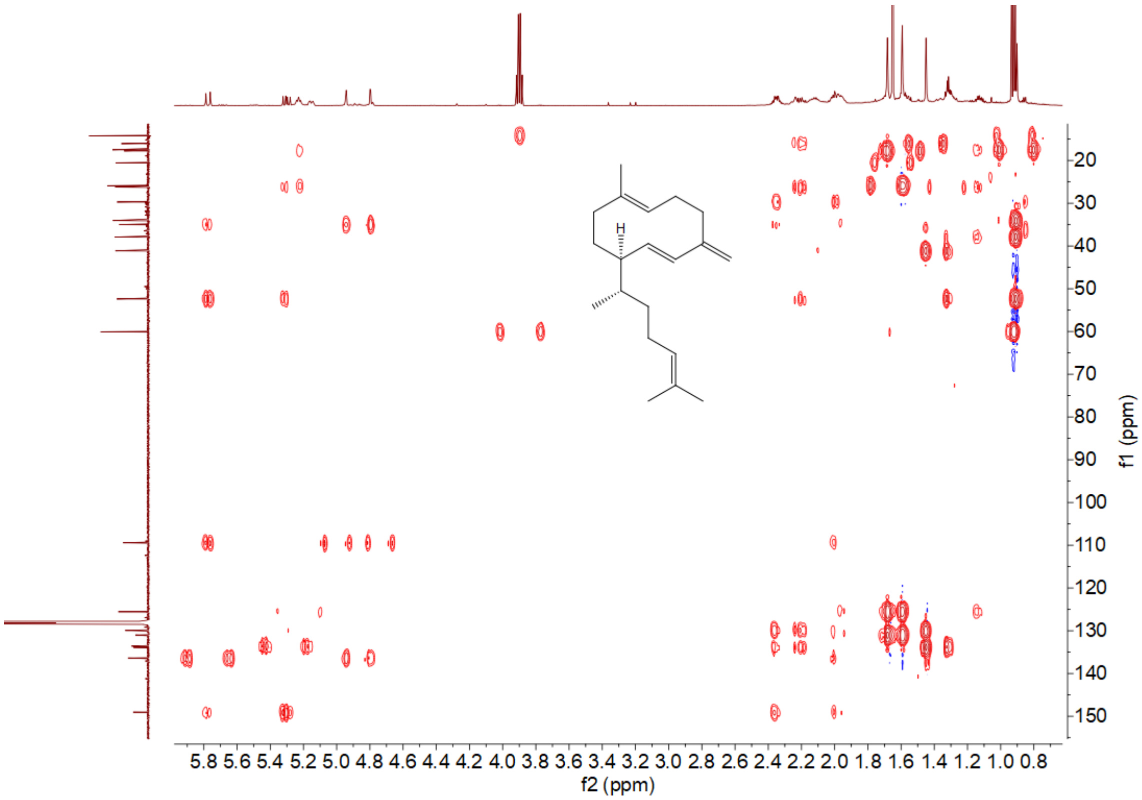

Figure S18. HMBC spectrum of compound **7** in C_6D_6.

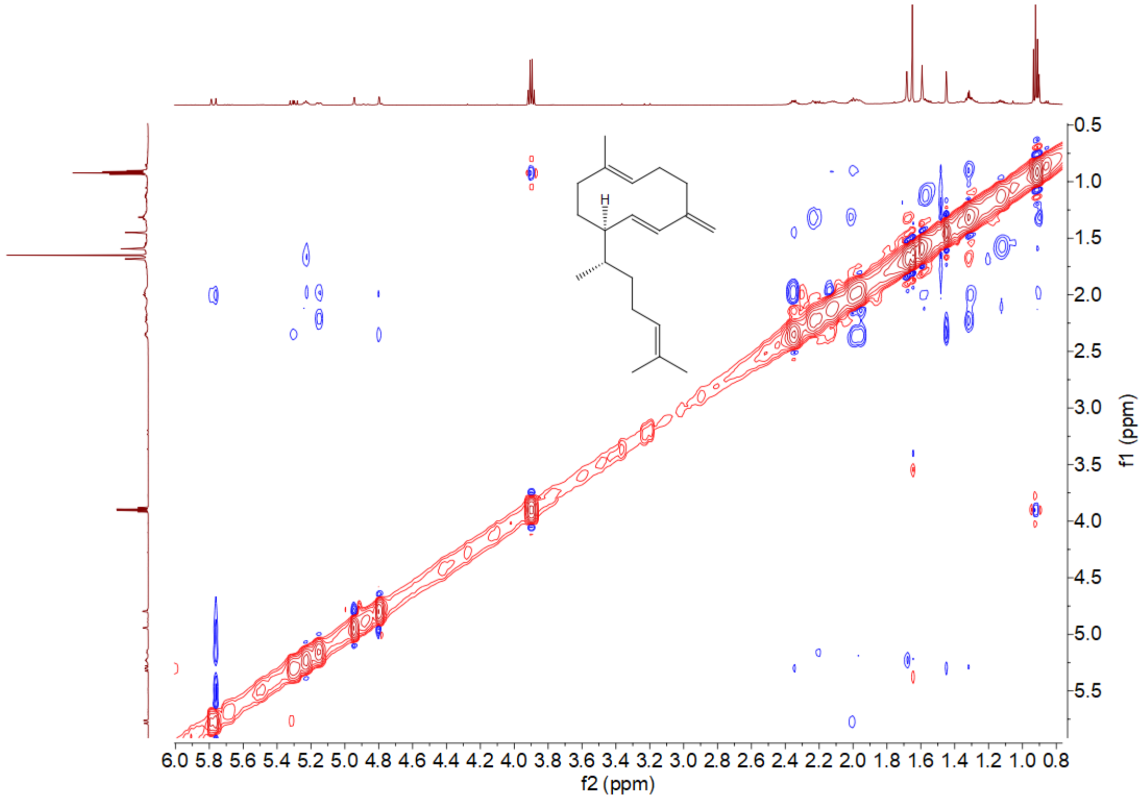

Figure S19. NOESY spectrum of compound **7** in C_6D_6.

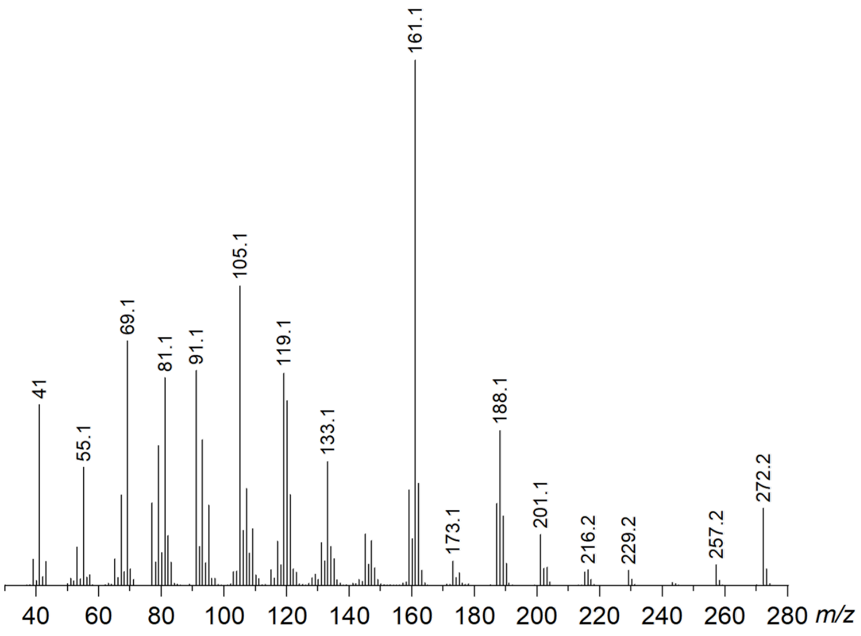

Figure S20. GC-MS spectrum of compound **7**.

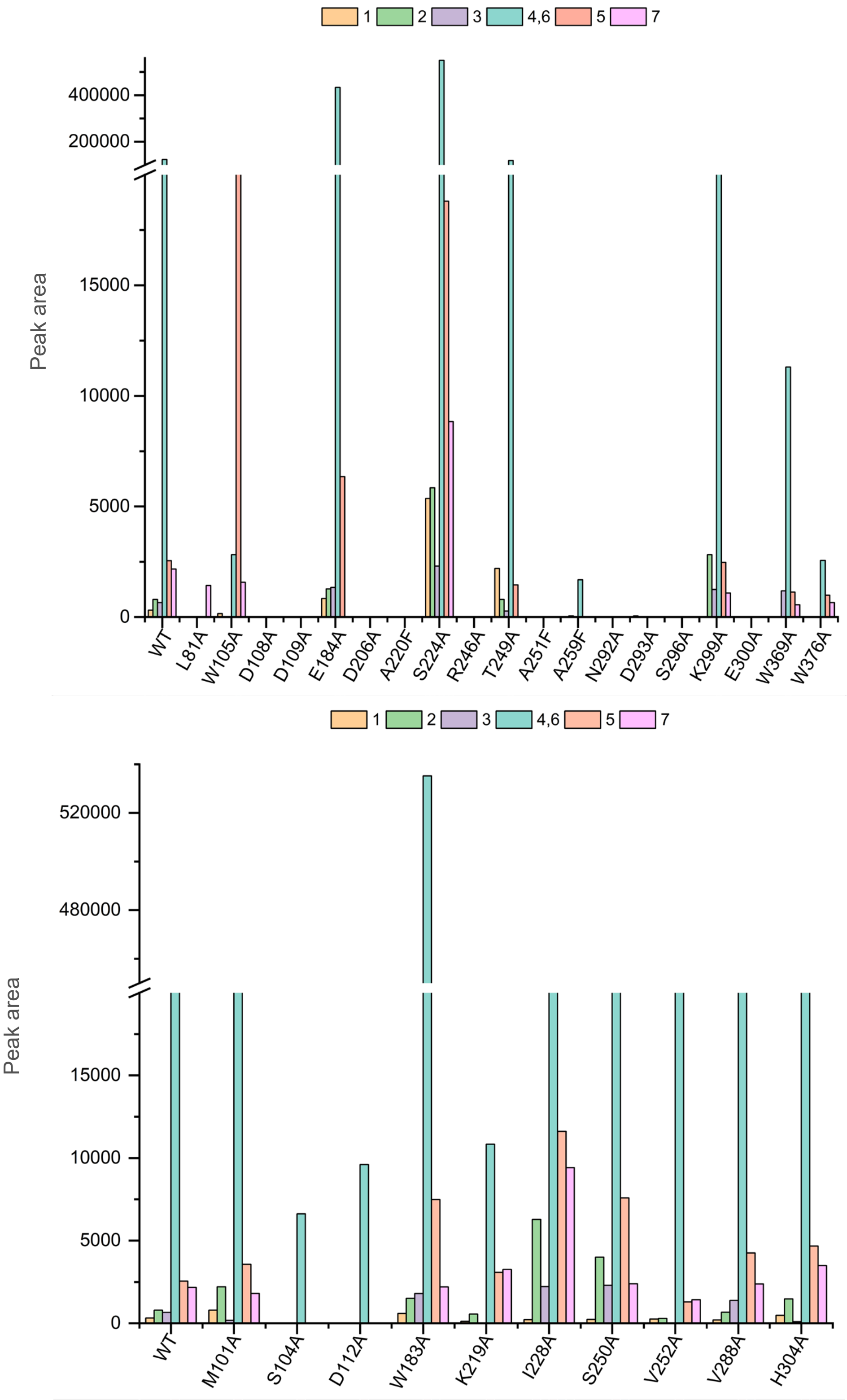

Figure S21. In vivo enzymatic activity comparison of wild-type *Hs*MTPSL1 and its mutants.

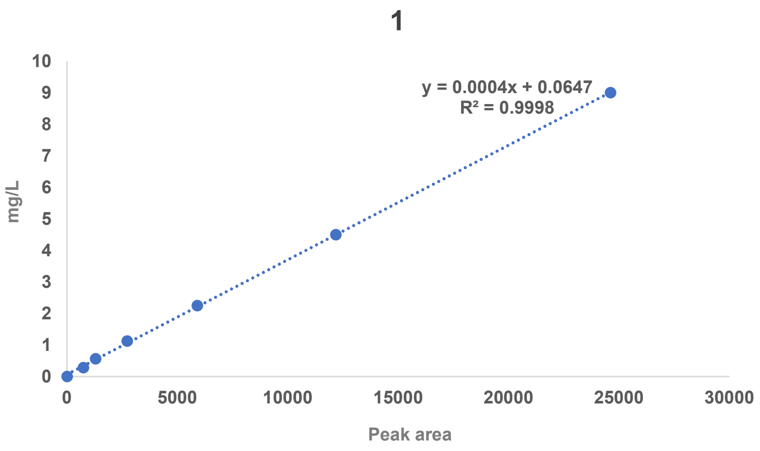

Figure S22. Standard curve of compound **1**.

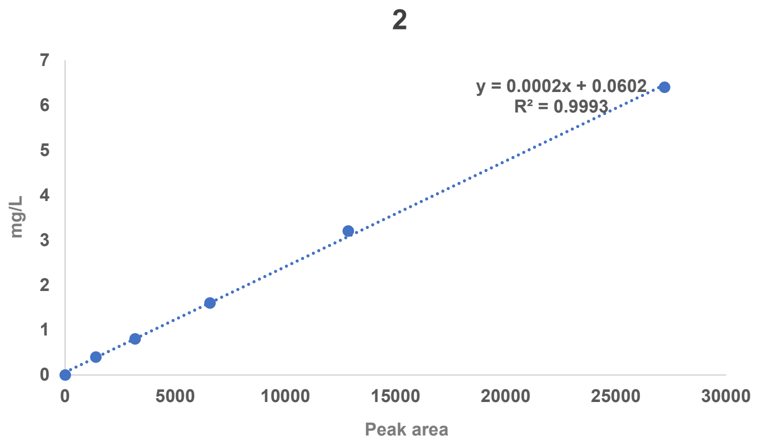

Figure S23. Standard curve of compound **2**.

```
          
HsMTPSL1  ................................................................................
SxSpS     ................................................................................
CrMTPSL3  MISTQTICLCSPGIPQPGICINSPVPLVSPLRRREGLNWSSGFQKGRGSSTSALKQESSFFSSAEDASKLKVQRSTTVQV

               1        10             20        30            40         50        60
HsMTPSL1  .....MVYECGQEHVAESPHESS......RRQDKLQLMKSYKVPEFIS....PFP.LRVHSMETEACKSNRNWLLRHSID
SxSpS     ............VDAVHGPEDGS...GPGSESGSASRPGAAQPPEIHC....PFP.SEMSPHAEHAEAHLDAWVARFEVV
CrMTPSL3  AAKHSVVSDLTGTKTVESVDPISPAQLPAQLPAQLGSPVDDQLPPLLSMIIDGFPPLEQSEYIHEAEILLKQYVERYPTI

               70        80        90          100       110       120       130       140
HsMTPSL1  KE.FSPDRFEKMLFCPALVTRIFPDASWKGLD...LSCKFMTLSWLMDDVFDETDINKSWEPSLSLSQEISLVMMSTFPK
SxSpS     RGTVARERFGRAGFA.QFAARTYPTADRACLD...LVADWFGWLFLVDDQLDDGRVGRDIDSARRAM.............
CrMTPSL3  TSL...ATYCRFGLCAAS....YAHPGVISLDRIVVMSINYALLFVLDDLFFDMRADFLL....................

                 150       160       170       180         190       200       210
HsMTPSL1  DQTIPECLMRVLELYASDRLEVIQNLAVDEILRNDNEAAA..KIFSGKLSLLGSAFRDVWDEIVAETNTESSLRIGRYFQ
SxSpS     .....DGLLRVLDR....................EGPAEG..ERPP.GEPPLAWALRDLWHRTASRATPAWRRRFTGHLA
CrMTPSL3  ............HQYGVSRDKLESPETIEEYIDHIGAVLGQQVQPCNPPPLIETMMSELGRNLMERSNPEWFRYYVDYFL

          220      230           240       250       260       270       280       290
HsMTPSL1  KAFISSLIQIQN....NKDKVIPSLDDYVLIRRGTSAVVPFVVIADLTDGVVLPEDIYANPQMQRLMDLVTDFVAWHNDI
SxSpS     ACLEAACWEAEN....RIAGVVPGEAEYIEQRRHTGAIYVCMDLIDIVGDLDLPEAVHAGEPFQAVLRASSDVVVWTNDW
CrMTPSL3  DCQRA.CIESEVDIIQGRNLYFQDVESYTVMRAANVAGKLTQVMTEFANDAYIPGILRVDPYFVKITTAASIHIGFVNDL

             300       310       320       330       340       350       360       370
HsMTPSL1  WSFKKEYLIHSDVHNLVFLISHHCNCSFEVAALTITKMLYVLCSEMEVAAKEVLKVTPPQHQHAASRYIVACRNWVSATD
SxSpS     YSLGKEMAL.GEYHNLVRVVAHARRLTLREALEH.TAAAISAETRRYLGHRERLLAAHPEHRAALTTCLAGMESWMRGNL
CrMTPSL3  FSYHRESAIEKNPRNLITVLMECEGMPFVQAAQTAVGLTNK.......YASEVVDLEAEAWNSTLRHYLVGIKAVLAGNI

             380
HsMTPSL1  DWHKKSLRYS..............................
SxSpS     DWSRATLRYRERERGGLPAYLEA....TLAPAGTEGGT..
CrMTPSL3  YFCLADMRYHHPDSA.FPELRDMTSSWKIVPRKKGQGLKL
```

Figure S24. Multiple sequence alignment of *Hs*MTPSL1 with *Sx*SpS and CrMTPSL3.

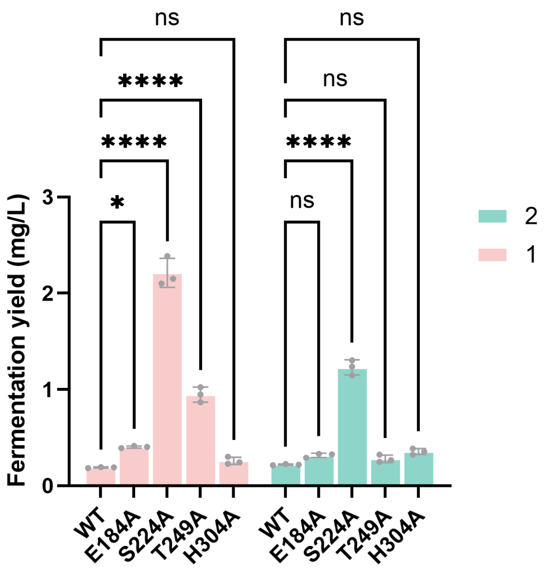

Figure S25. Mutants with increased yield of compounds **1** and **2**. Data are presented as mean ± SD (n = 3). **P* < 0.1, *****P* < 0.0001.

Supplementary Tables

Table S1. ^{1}H and ^{13}C NMR data for **7** in C_6D_6.

**No.**	***d*H (mult, *J* in Hz)**	***d*C (mult)**
1	5.30, dd (15.8, 10.0)	133.6, CH
2	5.77, d (15.8)	136.4, CH
3		149.0, C
4a	2.35, m	35.0, CH_2
4b	2.00, m	
5a	2.35, m	29.7, CH_2
5b	1.97, m	
6	5.15, m	129.9, CH
7		133.9, C
8a	2.23, m	41.1, CH_2
8b	2.23, m	
9a	1.97, m	26.2, CH_2
9b	1.31, m	
10	2.01, m	52.4, CH
11	1.31, m	37.9, CH
12a	1.57, m	34.0, CH_2
12b	1.13, m	
13a	2.12, m	26.3, CH_2
13b	2.12, m	
14	5.23, m	125.6, CH

Table S2. The yield of compounds **1** and **2** of wild-type *Hs*MTPSL1 and its mutants.

Type	1 (mg/L)	2 (mg/L)
WT	0.19	0.22
E184A	0.40	0.32
S224A	2.21	1.23
T249A	0.94	0.22
H304A	0.26	0.36

Table S3. MTPSLs from different species sources.

Gene name	Accession No. in GenBank
CrMTPSL3	APB88777.1
Mos-GOWD-MTPSL2	APB88775.1
Liv-IRBN-MTPSL2	APB88773.1
Liv-IRBN-MTPSL4	APB88774.1
Mon-GSXD-MTPSL3	APB88779.1
Mon-UJTT-MTPSL4	APB88780.1
Mon-YJJY-MTPSL1	APB88781.1
Mos-QKQO-MTPSL3	APB88776.1
ApMTPSL7	APB88778.1
ApMTPSL1	AVL27452.1
ApMTPSL2	AVL27462.1
ApMTPSL3	AVL27453.1
ApMTPSL4	AVL27454.1
ApMTPSL6	AVL27451.1
AaMTPSL1	AVL27456.1
AaMTPSL3	AVL27457.1
AaMTPSL4	AVL27458.1
AaMTPSL6	AVL27460.1
AaMTPSL7	AVL27461.1
SmMTPSL1	XP_002960898.1
SmMTPSL13	XP_002971857.1
SmMTPSL17	XP_024532655.1
SmMTPSL22	XP_002972952.1
SmMTPSL26	XP_024535054.1
SmMTPSL30	XP_002977567.1
MpMTPSL1	APP91786.1
MpMTPSL2	APP91787.1
MpMTPSL3	APP91788.1
MpMTPSL4	APP91789.1
MpMTPSL5	APP91790.1
MpMTPSL6	APP91791.1
MpMTPSL7	APP91792.1
MpMTPSL8	APP91793.1
MpMTPSL9	APP91797.1

Table S4. Protein sequence of *Hs*MTPSL1, *Sx*SpS and CrMTPSL3.

Name	Protein sequence
*Hs*MTPSL1	MVYECGQEHVAESPHESSRRQDKLQLMKSYKVPEFISPFPLRVHSMETEACKS<br>NRNWLLRHSIDKEFSPDRFEKMLFCPALVTRIFPDASWKGLDLSCKFMTLSWL<br>MDDVFDETDINKSWEPSLSLSQEISLVMMSTFPKDQTIPECLMRVLELYASDRL<br>EVIQNLAVDEILRNDNEAAAKIFSGKLSLLGSAFRDVWDEIVAETNTESSLRIG<br>RYFQKAFISSLIQIQNNKDKVIPSLDDYVLIRRGTSAVVPFVVIADLTDGVVLPE<br>DIYANPQMQRLMDLVTDFVAWHNDIWSFKKEYLIHSDVHNLVFLISHHCNC<br>SFEVAALTITKMLYVLCSEMEVAAKEVLKVTPPQHQHAASRYIVACRNWVSA<br>TDDWHKKSLRYS
*Sx*SpS	MVDAVHGPEDGSGPGSESGSASRPGAAQPPEIHCPFPSEMSPHAEHAEAHLD<br>AWVARFEVVRGTVARERFGRAGFAQFAARTYPTADRACLDLVADWFGWLF<br>LVDDQLDDGRVGRDIDSARRAMDGLLRVLDREGPAEGERPPGEPPLAWALR<br>DLWHRTASRATPAWRRRFTGHLAACLEAACWEAENRIAGVVPGEAEYIEQR<br>RHTGAIYVCMDLIDIVGDLDLPEAVHAGEPFQAVLRASSDVVVWTNDWYSL<br>GKEMALGEYHNLVRVVAHARRLTLREALEHTAAAISAETRRYLGHRERLLA<br>AHPEHRAALTTCLAGMESWMRGNLDWSRATLRYRERERGGLPAYLEATLAP<br>AGTEGGT
CrMTPSL3	MISTQTICLCSPGIPQPGICINSPVPLVSPLRRREGLNWSSGFQKGRGSSTSALK<br>QESSFFSSAEDASKLKVQRSTTVQVAAKHSVVSDLTGTKTVESVDPISPAQLPA<br>QLPAQLGSPVDDQLPPLLSMIIDGFPPLEQSEYIHEAEILLKQYVERYPTITSLAT<br>YCRFGLCAASYAHPGVISLDRIVVMSINYALLFVLDDLFFDMRADFLLHQYGV<br>SRDKLESPETIEEYIDHIGAVLGQQVQPCNPPPLIETMMSELGRNLMERSNPE<br>WFRYYVDYFLDCQRACIESEVDIIQGRNLYFQDVESYTVMRAANVAGKLTQV<br>MTEFANDAYIPGILRVDPYFVKITTAASIHIGFVNDLFSYHRESAIEKNPRNLIT<br>VLMECEGMPFVQAAQTAVGLTNKYASEVVDLEAEAWNSTLRHYLVGIKAVL<br>AGNIYFCLADMRYHHPDSAFPELRDMTSSWKIVPRKKGQGLKL

Table S5. Highly co-expressed CYP450 genes in *Huperzia serrata* transcriptome and their homologous genes.

Gene_id	correlation (r)	length	Homologous	sequence identity(%)	taxonomy
CL10099.Contig1	0.968373585	540	CYP782C1	91.92	Huperzia
CL10194.Contig18	0.967275729	214	CYP707A204	78.5	Ginkgo
CL7113.Contig3	0.94898463	611	CYP727E1	99.77	Huperzia
CL10099.Contig3	0.932370503	544	CYP782C1	85.45	Huperzia
CL1326.Contig3	0.924112922	330	CYP75B156	41.03	Panax
CL1326.Contig2	0.902180688	534	CYP781C1	.52.56	Lycopodium
Unigene73970	0.869998393	563	CYP4G258	90.76	Liposcelis
CL10194.Contig15	0.867718778	214	CYP707A204	78.5	Ginkgo
CL7113.Contig4	0.86442903	611	CYP727E1	99.77	Huperzia
CL11153.Contig2	0.833922838	445	CYP9EN2	68.09	Liposcelis
CL11162.Contig1	0.833922838	507	CYP710A89	73.87	Andreaea
CL2163.Contig20	0.833922838	320	CYP97A35	58.05	Calypogeia
Unigene60581	0.833922838	502	CYP4CC2	59.36	Liposcelis
Unigene61529	0.833922838	251	CYP51F1	86.06	Conserved
Unigene62189	0.833922838	200	CYP358B1	71.5	Liposcelis
Unigene62644	0.833922838	344	CYP6TP1	91.28	Liposcelis
Unigene64322	0.833922838	226	CYP4G259	86.73	Liposcelis
Unigene67595	0.833922838	507	CYP4C135	85.8	Liposcelis
Unigene67653	0.833922838	200	CYP4C134	70.5	Liposcelis
Unigene69449	0.833922838	434	CYP6CE12	67.59	Liposcelis
Unigene74292	0.833922838	270	CYP358B1	69.37	Liposcelis
Unigene74293	0.833922838	235	CYP4C134	77.97	Liposcelis
Unigene79620	0.833922838	206	CYP9EP1	68.6	Liposcelis
Unigene83544	0.833922838	422	CYP703A40	53.55	Dioon
Unigene69817	0.8328356	502	CYP4FD2	76.1	Liposcelis
Unigene62709	0.809476169	542	CYP301D1	71.32	Liposcelis
Unigene62688	0.805846463	463	CYP4SX1	74.08	Liposcelis

Table S6. Primer sequences used in this study.

Primer	Nucleotide Sequence (5′–3′)	Purpose
*Hs*MTPSL1-F	AGCAAATGGGTCGC**GGATCC**ATGGGTAGTGAAATTGCCGC	*Hs*MTPSL1 mutant amplification for expression in *E. coli*
*Hs*MTPSL1-R	TCGAGTGCGGCCGC**AAGCTT**TTAGCTATAGCGCAGGCTTT	
*Hs*MTPSL1-L81A-F	AAATGCTGTTTTGCCCGGCG**GCG**GTGACCCGCATTTTTCC	*Hs*MTPSL1 mutagenesis for L81A
*Hs*MTPSL1-L81A-R	GGAAAAATGCGGGTCAC**CGC**CGCCGGGCAAAACAGCATTT	
*Hs*MTPSL1-M101A-F	TGGATCTGAGCTGCAAATTT**GCG**ACCCTGAGCTGGCTGAT	*Hs*MTPSL1 mutagenesis for M101A
*Hs*MTPSL1-M101A-R	ATCAGCCAGCTCAGGGT**CGC**AAATTTGCAGCTCAGATCCA	
*Hs*MTPSL1-S104A-F	GCTGCAAATTTATGACCCTG**GCC**TGGCTGATGGATGATGT	*Hs*MTPSL1 mutagenesis for S104A
*Hs*MTPSL1-S104A-R	ACATCATCCATCAGCCA**GGC**CAGGGTCATAAATTTGCAGC	
*Hs*MTPSL1-W105A-F	AATTTATGACCCTGAGC**GCG**CTGATGGATGATGTGTTTGA	*Hs*MTPSL1 mutagenesis for W105A
*Hs*MTPSL1-W105A-R	TCAAACACATCATCCATCAG**CGC**GCTCAGGGTCATAAATT	
*Hs*MTPSL1-D108A-F	CCCTGAGCTGGCTGATG**GCT**GATGTGTTTGATGAAACCGA	*Hs*MTPSL1 mutagenesis for D108A
*Hs*MTPSL1-D108A-R	TCGGTTTCATCAAACACATC**AGC**CATCAGCCAGCTCAGGG	
*Hs*MTPSL1-D109A-F	CCCTGAGCTGGCTGATGGAT**GCT**GTGTTTGATGAAACCGA	*Hs*MTPSL1 mutagenesis for D109A
*Hs*MTPSL1-D109A-R	TCGGTTTCATCAAACAC**AGC**ATCCATCAGCCAGCTCAGGG	
*Hs*MTPSL1-D112A-F	TGATGGATGATGTGTTT**GCT**GAAACCGATATTAACAAAAG	*Hs*MTPSL1 mutagenesis for D112A
*Hs*MTPSL1-D112A-R	CTTTTGTTAATATCGGTTTC**AGC**AAACACATCATCCATCA	
*Hs*MTPSL1-W183A-F	ACGAAGCGGCCGCGAAAATT**GCT**AGCGGCAAACTGAGCCT	*Hs*MTPSL1 mutagenesis for W183A
*Hs*MTPSL1-W183A-R	AGGCTCAGTTTGCCGCT**AGC**AATTTTCGCGGCCGCTTCGT	
*Hs*MTPSL1-E184A-F	CGGCCGCGAAAATTTTT**GCC**GGCAAACTGAGCCTGCTGGG	*Hs*MTPSL1 mutagenesis for E184A
*Hs*MTPSL1-E184A-R	CCCAGCAGGCTCAGTTTGCC**GGC**AAAAATTTTCGCGGCCG	
*Hs*MTPSL1-D206A-F	ATGAAATTGTGGCGGAAACC**GCC**ACCGAAAGCAGCCTGCG	*Hs*MTPSL1 mutagenesis for D206A
*Hs*MTPSL1-D206A-R	CGCAGGCTGCTTTCGGT**GGC**GGTTTCCGCCACAATTTCAT	
*Hs*MTPSL1-K219A-F	GCATTGGCCGCTATTTTCAG**GCA**GCGTTTATTAGCAGCCT	*Hs*MTPSL1

*Hs*MTPSL1-K219A-R	AGGCTGCTAATAAACGC**TGC**CTGAAAATAGCGGCCAATGC	mutagenesis for K219A
*Hs*MTPSL1-A220F-F	TTGGCCGCTATTTTCAGAAA**TTT**TTTATTAGCAGCCTGAT	*Hs*MTPSL1 mutagenesis for A220F
*Hs*MTPSL1-A220F-R	ATCAGGCTGCTAATAAA**AAA**TTTCTGAAAATAGCGGCCAA	
*Hs*MTPSL1-S224A-F	TTCAGAAAGCGTTTATTAGC**GCC**CTGATTCAGATTCAGAA	*Hs*MTPSL1 mutagenesis for S224A
*Hs*MTPSL1-S224A-R	TTCTGAATCTGAATCAG**GGC**GCTAATAAACGCTTTCTGAA	
*Hs*MTPSL1-I228A-F	TTATTAGCAGCCTGATTCAG**GCT**CAGAACAACAAAGATAA	*Hs*MTPSL1 mutagenesis for I228A
*Hs*MTPSL1-I228A-R	TTATCTTTGTTGTTCTG**AGC**CTGAATCAGGCTGCTAATAA	
*Hs*MTPSL1-R246A-F	ATGATTATGTGCTGATT**GCC**CGCGGCACGAGCGCGGTGGT	*Hs*MTPSL1 mutagenesis for R246A
*Hs*MTPSL1-R246A-R	ACCACCGCGCTCGTGCCGCG**GGC**AATCAGCACATAATCAT	
*Hs*MTPSL1-T249A-F	ATGTGCTGATTCGCCGCGGC**GCG**AGCGCGGTGGTGCCGTT	*Hs*MTPSL1 mutagenesis for T249A
*Hs*MTPSL1-T249A-R	AACGGCACCACCGCGCT**CGC**GCCGCGGCGAATCAGCACAT	
*Hs*MTPSL1-S250A-F	TGCTGATTCGCCGCGGCACG**GCC**GCGGTGGTGCCGTTTGT	*Hs*MTPSL1 mutagenesis for S250A
*Hs*MTPSL1-T250A-R	ACAAACGGCACCACCGC**GGC**CGTGCCGCGGCGAATCAGCA	
*Hs*MTPSL1-A251F-F	TGATTCGCCGCGGCACGAGC**TTT**GTGGTGCCGTTTGTGGT	*Hs*MTPSL1 mutagenesis for A251F
*Hs*MTPSL1-A251F-R	ACCACAAACGGCACCAC**AAA**GCTCGTGCCGCGGCGAATCA	
*Hs*MTPSL1-V252A-F	TTCGCCGCGGCACGAGCGCG**GCG**GTGCCGTTTGTGGTGAT	*Hs*MTPSL1 mutagenesis for V252A
*Hs*MTPSL1-V252A-R	ATCACCACAAACGGCAC**CGC**CGCGCTCGTGCCGCGGCGAA	
*Hs*MTPSL1-A259F-F	TGGTGCCGTTTGTGGTGATT**TTT**GATCTGACCGATGGCGT	*Hs*MTPSL1 mutagenesis for A259F
*Hs*MTPSL1-A259F-R	ACGCCATCGGTCAGATC**AAA**AATCACCACAAACGGCACCA	
*Hs*MTPSL1-V288A-F	TGGATCTGGTGACCGATTTT**GCG**GCGTGGCATAACGATAT	*Hs*MTPSL1 mutagenesis for V288A
*Hs*MTPSL1-V288A-R	ATATCGTTATGCCACGC**CGC**AAAATCGGTCACCAGATCCA	
*Hs*MTPSL1-H291A-F	CCGATTTTGTGGCGTGG**GCT**AACGATATTTGGAGCTTTAA	*Hs*MTPSL1 mutagenesis for(H291A)
*Hs*MTPSL1-H291A-R	TTAAAGCTCCAAATATCGTT**AGC**CCACGCCACAAAATCGG	
*Hs*MTPSL1-N292A-F	CCGATTTTGTGGCGTGGCAT**GCC**GATATTTGGAGCTTTAA	*Hs*MTPSL1 mutagenesis for N292A
*Hs*MTPSL1-N292A-R	TTAAAGCTCCAAATATC**GGC**ATGCCACGCCACAAAATCGG	

*Hs*MTPSL1-D293A-F	CCGATTTTGTGGCGTGGCATAAC**GCT**ATTTGGAGCTTTAA	*Hs*MTPSL1 mutagenesis for D293A
*Hs*MTPSL1-D293A-R	TTAAAGCTCCAAAT**AGC**GTTATGCCACGCCACAAAATCGG	
*Hs*MTPSL1-S296A-F	CGTGGCATAACGATATTTGG**GCC**TTTAAAAAAGAATATCT	*Hs*MTPSL1 mutagenesis for S296A
*Hs*MTPSL1-S296A-R	AGATATTCTTTTTTAAA**GGC**CCAAATATCGTTATGCCACG	
*Hs*MTPSL1-K299A-F	GATATTTGGAGCTTTAAA**GCA**GAATATCTGATTCATAGCG	*Hs*MTPSL1 mutagenesis for K299A
*Hs*MTPSL1-K299A-R	CGCTATGAATCAGATATTC**TGC**TTTAAAGCTCCAAATATC	
*Hs*MTPSL1-E300A-F	ATATTTGGAGCTTTAAAAAA**GCA**TATCTGATTCATAGCGA	*Hs*MTPSL1 mutagenesis for E300A)
*Hs*MTPSL1-E300A-R	TCGCTATGAATCAGATA**TGC**TTTTTTAAAGCTCCAAATAT	
*Hs*MTPSL1-H304A-F	AAAAAGAATATCTGATT**GCT**AGCGATGTGCATAACCTGGT	*Hs*MTPSL1 mutagenesis for H304A
*Hs*MTPSL1-H304A-R	ACCAGGTTATGCACATCGCT**AGC**AATCAGATATTCTTTTT	
*Hs*MTPSL1-W369A-F	TTGTGGCGTGCCGCAAC**GCG**GTGAGCGCGACCGATGATTG	*Hs*MTPSL1 mutagenesis for W369A
*Hs*MTPSL1-W369A-R	CAATCATCGGTCGCGCTCAC**CGC**GTTGCGGCACGCCACAA	
*Hs*MTPSL1-W376A-F	TGAGCGCGACCGATGAT**GCG**CATAAAAAAAGCCTGCGCTA	*Hs*MTPSL1 mutagenesis for W376A
*Hs*MTPSL1-W376A-R	TAGCGCAGGCTTTTTTTATG**CGC**ATCATCGGTCGCGCTCA	
*Hs*MTPSL1-H377A-F	TGAGCGCGACCGATGATTGG**GCT**AAAAAAAGCCTGCGCTA	*Hs*MTPSL1 mutagenesis for H377A
*Hs*MTPSL1-H377A-R	TAGCGCAGGCTTTTTTT**AGC**CCAATCATCGGTCGCGCTCA	

Table S7. Plasmids used in this study.

Plasmid	Description	Source (Reference)
pCDF-MKI4	Kinase-based system for GGPP production	[5]
pET28a	General plasmid for cloning and protein production	Weidibio, Shanghai, China
pET28a- HSMTPSL1-L81A	pET28a harboring HsMTPSL1(L81A)	this study
pET28a- HSMTPSL1-M101A	pET28a harboring HsMTPSL1(M101A)	this study
pET28a- HSMTPSL1-S104A	pET28a harboring HsMTPSL1(S104A)	this study
pET28a- HSMTPSL1-W105A	pET28a harboring HsMTPSL1(W105A)	this study
pET28a- HSMTPSL1-D108A	pET28a harboring HsMTPSL1(D108A)	this study
pET28a- HSMTPSL1-D109A	pET28a harboring HsMTPSL1(D109A)	this study
pET28a- HSMTPSL1-D112A	pET28a harboring HsMTPSL1(D112A)	this study
pET28a- HSMTPSL1-W183A	pET28a harboring HsMTPSL1(W183A)	this study
pET28a- HSMTPSL1-E184A	pET28a harboring HsMTPSL1(E184A)	this study
pET28a- HSMTPSL1-D206A	pET28a harboring HsMTPSL1(D206A)	this study
pET28a- HSMTPSL1-K219A	pET28a harboring HsMTPSL1(K219A)	this study
pET28a- HSMTPSL1-A220F	pET28a harboring HsMTPSL1(A220F)	this study
pET28a- HSMTPSL1-S224A	pET28a harboring HsMTPSL1(S224A)	this study
pET28a- HSMTPSL1-I228A	pET28a harboring HsMTPSL1(I228A)	this study
pET28a- HSMTPSL1-R246A	pET28a harboring HsMTPSL1(R246A)	this study
pET28a- HSMTPSL1-T249A	pET28a harboring HsMTPSL1(T249A)	this study
pET28a- HSMTPSL1-S250A	pET28a harboring HsMTPSL1(S250A)	this study
pET28a- HSMTPSL1-A251F	pET28a harboring HsMTPSL1(A251F)	this study
pET28a- HSMTPSL1-V252A	pET28a harboring HsMTPSL1(V252A)	this study
pET28a- HSMTPSL1-A259F	pET28a harboring HsMTPSL1(A259F)	this study
pET28a- HSMTPSL1-V288A	pET28a harboring HsMTPSL1(V288A)	this study
pET28a- HSMTPSL1-H291A	pET28a harboring HsMTPSL1H291A)	this study

pET28a- HSMTPSL1-N292A	pET28a harboring HsMTPSL1(N292A)	this study
pET28a- HSMTPSL1-D293A	pET28a harboring HsMTPSL1(D293A)	this study
pET28a- HSMTPSL1-S296A	pET28a harboring HsMTPSL1(S296A)	this study
pET28a- HSMTPSL1-K299A	pET28a harboring HsMTPSL1(K299A)	this study
pET28a- HSMTPSL1-E300A	pET28a harboring HsMTPSL1(E300A)	this study
pET28a- HSMTPSL1-H304A	pET28a harboring HsMTPSL1(H304A)	this study
pET28a- HSMTPSL1-W369A	pET28a harboring HsMTPSL1(W369A)	this study
pET28a- HSMTPSL1-W376A	pET28a harboring HsMTPSL1(W376A)	this study
pET28a- HSMTPSL1-H377A	pET28a harboring HsMTPSL1(H377A)	this study

REFERENCES

1 Rinkel, J.; Lauterbach, L.; Dickschat, J.S. Spata-13,17-diene synthase-An enzyme with sesqui-, di-, and sesterterpene synthase activity from *Streptomyces xinghaiensis*. *Angew. Chem., Int. Ed.* **2017,** 56, (51), 16385–16389.

2 Kolesnikova, S.A.; Kalinovsky, A.I.; Fedorov, S.N.; Shubina, L.K.; Stonik, V.A. Diterpenes from the Far-eastern brown alga *Dictyota dichotoma*. *Phytochemistry* **2006,** 67, (19), 2115–2119.

3 Cesati, de Armas, J.; Hoveyda, A.H. Enantioselective total synthesis of erogorgiaene: applications of asymmetric Cu-catalyzed conjugate additions of alkylzincs to acyclic enones. *J. Am. Chem. Soc.* **2004,** 126, (1), 96–101.

4 Rinkel, J.; Rabe, P.; Chen, X.; Köllner, T.G.; Chen, F.; Dickschat, J.S. Mechanisms of the diterpene cyclases beta-pinacene synthase from *Dictyostelium discoideum* and hydropyrene synthase from *Streptomyces clavuligerus*. *Chemistry* **2017,** 23, (44), 10501–10505.

5 Chen, B.; Mao, J.J.; Xu, K.W.; Liu, L.J.; Lin, W.; Guo, Y.-W.; Wu, R.B.; Wang, C.Y.; Xu, B.F. Mining coral-derived terpene synthases and mechanistic studies of the coral biflorane synthase. *Sci. Adv.* **2025,** 11, (9), eadv0805.

6 Li, L.; Sheng, L.; Wang, C.-Y.; Zhou, Y.-B.; Huang, H.; Li, X.-B.; Li, J.; Mollo, E.; Gavagnin, M.; Guo, Y.-W. Diterpenes from the Hainan soft coral *Lobophytum cristatum* Tixier-Durivault. *J. Nat. Prod.* **2011,** 74, (10), 2089–2094.
